# Supervised Versus Unsupervised Exercise for the Improvement of Physical Function and Well-Being Outcomes in Older Adults: A Systematic Review and Meta-analysis of Randomized Controlled Trials

**DOI:** 10.1007/s40279-024-02024-1

**Published:** 2024-04-22

**Authors:** Paola Gómez-Redondo, Pedro L. Valenzuela, Javier S. Morales, Ignacio Ara, Asier Mañas

**Affiliations:** 1 GENUD Toledo Research Group, Faculty of Sports Sciences, Universidad de Castilla-La Mancha, Avda. Carlos III s/n, 45071 Toledo, Spain; 2 CIBER On Frailty and Healthy Aging, Instituto de Salud Carlos III, Madrid, Spain; 3 Instituto de Investigación Sanitaria de Castilla-La Mancha (IDISCAM), Junta de Comunidades de Castilla-La Mancha (JCCM), Toledo, Spain; 4 Physical Activity and Health Research Group (PaHerg), Research Institute of Hospital, 12 de Octubre (imas12), Madrid, Spain; 5 Biology Systems Department, University of Alcalá, Madrid, Spain; 6 MOVE-IT Research Group, Department of Physical Education, Faculty of Education Sciences, University of Cadiz, Cadiz, Spain; 7 Biomedical Research and Innovation Institute of Cádiz (INiBICA) Research Unit, Puerto Real University Hospital, University of Cadiz, Cadiz, Spain; 8 Center UCM-ISCIII for Human Evolution and Behavior, 28029 Madrid, Spain; 9 Faculty of Education, Complutense University of Madrid, 28040 Madrid, Spain

## Abstract

**Background:**

Unsupervised exercise intervention (UNSUP) appears to be a practical and beneficial strategy for older adults, although its feasibility and effectiveness compared to supervised exercise intervention (SUP) remains unknown. We aimed to compare the safety, attendance/adherence rates, and effectiveness of SUP versus UNSUP on physical function and well-being outcomes in older adults.

**Methods:**

A systematic search was conducted in PubMed, Web of Science, CINAHL, SPORTDiscus, and APA PsycINFO up to September 2022 for randomized controlled trials comparing SUP versus UNSUP in older adults (≥ 60 years). Safety and attendance/adherence rates were registered as indicators of feasibility, and meta-analyses were performed for physical function and well-being outcomes. Sub-analyses were performed for those studies that applied a similar intervention in both groups and for those studies where participants performed ≥ 66% of the sessions in the assigned condition.

**Results:**

Thirty-four studies were included (*n* = 2830). No serious adverse events were reported, with similar attendance rates (81%) for both SUP and UNSUP. Compared with UNSUP, SUP induced significant higher benefits on knee extension strength (standardized mean difference (SMD) = 0.18, *p* = 0.002), sit-to-stand test (STS, SMD = 0.25, *p* = 0.050), timed-up-and-go test (TUG, SMD = 0.21, *p* = 0.035), usual gait speed (SMD = 0.29, *p* = 0.026), lean mass (mean difference = 1.05 kg, *p* < 0.001) and health-related quality of life (HRQoL, SMD = 0.21, *p* = 0.035), albeit only knee extension strength remained significant in sensitivity analyses. Sub-analyses revealed superior benefits of SUP on knee extension strength when only considering those studies that applied a similar intervention in both SUP and UNSUP groups. However, no significant benefits were found for the remaining outcomes. Beneficial effects of SUP over UNSUP were also observed for knee extension strength, STS, functional reach test, TUG, usual gait speed, lean mass, and HRQoL when separately analyzing those studies in which participants performed ≥ 66% of the sessions in the assigned condition.

**Conclusions:**

Current evidence suggests that both SUP and UNSUP programs are safe and could exert benefits on physical function and HRQoL. However, despite being associated with similar attendance rates, SUP might offer some additional benefits, although further high-quality research (i.e., accounting for confounding factors such as presence of supervised sessions in UNSUP or vice versa, as well as equating the exercise dose) is necessary to confirm these findings.

**PROSPERO Registration Number:**

CRD42022326420.

**Supplementary Information:**

The online version contains supplementary material available at 10.1007/s40279-024-02024-1.

## Key Points

**Table:** 

The present systematic review and meta-analysis of randomized controlled trials (34 studies, *n* = 2830 participants aged ≥ 60 years) shows that supervised exercise intervention (SUP) brings significantly superior benefits compared with unsupervised exercise intervention (UNSUP) on physical function and well-being outcomes.
Significant benefits of SUP over UNSUP were still found for knee extension strength in those studies that applied a similar exercise program in both groups. In addition, greater benefits of SUP were observed compared with UNSUP when participants performed at least 66% of the training sessions in the assigned condition.
Given that both programs are safe and show similar attendance rates, UNSUP could represent a cost-effective tool for improving physical function and well-being in this population when SUP is not feasible.

## Introduction

The population aged 60 years or over is rapidly growing, with the number of older adults worldwide expected to reach 1.4 billion in 2030 and 3.1 billion by 2100 [1]. This epidemiological shift is accompanied by a concomitant increase in the so-called aging-related diseases, notably frailty [2, 3]. Consequently, efforts are needed to attenuate aging-related deterioration and its associated burden.

Strong evidence supports the benefits of regular physical exercise for attenuating aging-related multisystem deterioration [4, 5]. Despite being overall beneficial, supervised exercise intervention (SUP)—the most widely analyzed type of intervention in the scientific literature—might have some drawbacks, as older adults can face difficulties in joining these interventions due to variables such as physical or financial constraints, low availability of facilities, weather conditions, distance from home, time commitments, the intimidating gym environment or, more recently, the lockdowns imposed by the COVID-19 pandemic [6]. In this context, unsupervised exercise intervention (UNSUP) appears to be a practical and potentially effective alternative [7, 8]. Indeed, a recent meta-analysis by our research group concluded that, despite being associated with modest adherence rates (67%), UNSUP might be effective for improving some important physical fitness outcomes in older adults compared with performing no exercise [9]. Similar results were reported by a recent meta-analysis that found a beneficial effect of UNSUP on physical fitness measures in healthy older adults [10].

There is therefore evidence to suggest that UNSUP is effective for improving physical fitness and overall health in older adults. It is worth noting, however, that controversy exists as to whether UNSUP might provide comparable benefits to those provided by SUP [11, 12]. A meta-analysis by Fisher et al. [12] reported that supervised strength training induces small benefits on muscle strength compared to unsupervised training in adolescents and adults, with little or no additional benefits on body composition. On the other hand, a meta-analysis by Lacroix et al. [13] found greater benefits on muscle strength/power and balance with SUP compared with UNSUP in healthy older adults. Nevertheless, the abovementioned results can be confounded by several factors, notably that most studies comparing SUP versus UNSUP have performed different interventions in each group (e.g., both groups did not perform the same type of training or the exercises performed were not comparable) [14–16]. Moreover, it is noteworthy that groups are frequently classified as SUP despite including unsupervised sessions, and conversely, the designation of UNSUP is sometimes applied to groups that also receive some supervised sessions [17, 18].

In this context, the aim of the present systematic review and meta-analysis of randomized controlled trials (RCTs) was to compare the safety, attendance/adherence rates, and effectiveness of SUP versus UNSUP on physical function and well-being measures in older adults, as well as to confirm whether differences are still present after accounting for potential confounding factors.

## Methods

This systematic review and meta-analysis was reported according to the PRISMA (Preferred Reporting Items for Systematic Revies and Meta-Analyses) statement [19] and is conducted following the principles proposed elsewhere [20]. The review protocol was registered in PROSPERO (CRD42022326420).

### Data Sources and Search Strategies

A systematic search was performed in the electronic databases PubMed, Web of Science, CINAHL, SPORTDiscus, and APA PsycINFO for relevant articles written in English (from inception to 4 September 2022). Screening of the articles was performed independently by two authors (AM, PGR). The complete search strategy is summarized in Online Supplementary Material (OSM) Table S1. The search was supplemented by a manual review of reference lists from included primary studies and review articles to find additional studies on the subject.

### Study Selection

Eligibility criteria are reported according to the Population, Intervention, Comparison, Outcome and Study design (PICOS) approach [21]. The review was limited to studies that met the criteria shown in OSM Table S2.

Studies were first retrieved and preliminarily screened by title and abstract, and the full texts of those studies that met the inclusion criteria were assessed (AM, PGR). Disagreements between authors were resolved through consensus or after consultation with a third reviewer (PLV).

An exercise session was considered supervised when participants received synchronous supervision from a professional (e.g., an initial instructional session showing the exercises to ensure the correct technique, or individual/group supervised sessions conducted over the intervention period) whether face-to-face or videocall format. On the other hand, an exercise session was considered unsupervised if it did not include synchronous supervision by a sports scientist (e.g.*,* phone calls asking about the exercises performed, assessing exercise frequency).

In the sub-analysis performed in this study, a supervised exercise group (SUP) was considered applicable if most training sessions performed had synchronous supervision by a sports scientist (*i.e.,* at least 66% of the training sessions were supervised). An unsupervised exercise group (UNSUP) was considered applicable if the main part of the training sessions was conducted without synchronous supervision by an exercise professional (i.e., at least 66% of the training sessions were conducted without real-time supervision). Two independent reviewers (JSM, PGR) checked information from the included studies to calculate ratios in Table 1. In cases of disagreement, a third author (PLV) was consulted for clarification. This 66% cut-off has been applied in previous systematic reviews and meta-analyses comparing supervised and unsupervised exercise training [13].

**Table 1:** Supervised training sessions ratio over total number of training sessions in SUP and UNSUP groups

Study	Number of training sessions in SUP groups	Number of supervised sessions in SUP groups	SUP groups ratio	Number of training sessions in UNSUP groups	Number of supervised sessions in UNSUP groups	UNSUP groups ratio
Almeida et al. [24]	52	52	1	52	9	0.17
Bieler et al. [18]	52	35	0.67	52	1	0.02
Bieler et al. [26]	52	35	0.67	52	1	0.02
Bieler et al. [25]	52	35	0.67	52	1	0.02
Bittar et al. [27]	104	104	1	104	0	0
Boshuizen et al. [49]	30	20	0.67	30	10	0.33
Brown et al. [16]	36	36	1	36	3	0.08
Cecchi et al. [32]	26	26	1	26	0	0
Costa et al. [17]	36	36	1	36	12	0.33
Cyarto et al. [28]	40	40	1	40	8	0.2
Cyarto et al. [29]	40	40	1	40	8	0.2
Donat et al. [30]	24	24	1	24	3	0.13
Ebersbach et al. [31]	16	16	1	16	1	0.06
Gardner et al. [33]	36	36	1	36	7	0.19
Gardner et al. [34]	36	36	1	36	0	0
Hinman et al. [35]	12	12	1	12	1	0.08
Horton et al. [36]	49	14	0.29	49	0	0
Iliffe et al. [37]	72	24	0.33	72	0	0
Kakkos et al. [48]	78	78	1	78	0	0
Karahan et al. [50]	30	30	1	30	1	0.03
Lacroix et al. [55]	36	24	0.67	36	0	0
Lindemann et al. [38]	16	16	1	16	2	0.13
Mickle et al. [39]	36	36	1	36	0	0
Morrison et al. [40]	36	36	1	36	1	0.03
Nai-Hsin et al. [41]	39	39	1	39	1	0.03
Opdenacker et al. [54]	132	132	1	132	8	0.06
Perez-Dominguez et al. [43]	48	48	1	48	16	0.33
Sandberg et al. [56]	78	78	1	78	2	0.03
Sian et al. [47]	12	12	1	12	1	0.08
Tsekoura et al. [44]	24	24	1	24	4	0.17
Van Roie et al. [15]	132	132	1	132	8	0.06
Watanabe et al. [45]	48	12	0.25	48	0	0
Watson et al. [14]	69	69	1	69	0	0
Wu et al. [46]	45	45	1	45	0	0

*SUP* supervised exercise interventions, *UNSUP* unsupervised exercise interventions

^*^Ratio was calculated based on supervised sessions/total number of sessions

### Outcomes Assessment

Safety included the number of adverse events (e.g.*,* injury, pain, discomfort, worsening of an existing condition) as well as the number of falls during the intervention period.

Attendance rates refer to whether or not the participant carries out the exercise sessions. On the other hand, adherence refers to whether the participant, in addition to attending the exercise sessions, has achieved the intended objectives (i.e.*,* volume, intensity, duration, exercises) [22].

To evaluate effectiveness, studies included should assess at least one of the following health-related endpoints: (1) muscle strength (e.g.*,* knee extension strength, handgrip strength), (2) balance (e.g., one leg stance, tandem stance) (3) physical performance (e.g., timed-up-and-go test, maximum gait speed), (4) body composition (e.g., body fat, lean mass), or (5) health-related quality of life (e.g., European Quality of Life 5 Dimensions (EQ-5D-5L), 36-Item Short-Form Health Survey). If studies reported multiple variables within one of the endpoints categories, all variables were included. Only those variables that were included in at least three studies were used for meta-analysis.

### Data Extraction

Two authors (JSM, PGR) independently extracted the following data from each study: participants’ characteristics, characteristics of the exercise interventions, attendance and adherence rates, outcomes assessed, and main results. This information was reviewed by a third author (AM) to ensure accuracy and completeness. Data comparing baseline and post-intervention assessments were used. We contacted the authors when studies reported the calculated change. Data were extracted, when available, as mean, standard deviation (SD), and number of participants per group. When data were provided as intervention effects and/or using other measures of dispersion (e.g., standard error, 95% confidence interval (CI)), the required information was estimated following the guidelines reported elsewhere [23]. When available, we used the results based on “intention-to-treat” analyses. We had to contact the authors of 30 studies [15, 16, 18, 24–50] because the required data were not reported. Of these, the authors of 11 studies [15, 18, 25, 26, 31, 36, 37, 39, 44, 49, 50] provided the required information.

### Quality Assessment

Two authors (AM, PGR) independently assessed the methodological quality of the included studies with the Tool for the assEssment of Study qualiTy and reporting in Exercise (TESTEX) scale [51]. This is a 15-point scale specifically designed for use in exercise training studies, including 5 points for study quality and 10 points for reporting. Thus, the quality of the studies was classified according to their total TESTEX score as “high” (≥ 12 points), “good” (7–11), or “low” (≤ 6). All the studies were used for data synthesis independently of their methodological quality. A third author (JSM) resolved any potential disagreement.

### Statistical Analysis

A random-effects meta-analysis (DerSimonian and Laird method) was performed when at least three studies assessed a given outcome. The pooled standardized mean difference (SMD, post- minus pre-intervention data) between interventions was computed along with the 95%CI, and if the studies reported a given outcome using the same measurement units (e.g., kg, meters), the absolute mean difference (MD) was computed. A conservative correlation coefficient (Pearson’s *r*-value) of 0.7 between pre- and post-intervention data was used for the computation of the within-group SD, and sensitivity analyses with an *r*-value of 0.2 and 0.5 were performed when a significant result was found (not reported unless results became non-significant) [52]. When a study provided effect sizes separately for a given outcome divided in different sub-scales (i.e., health-related quality of life (HRQoL) divided into its different subdomains), results for that study were combined following a conservative approach by using a random-effects model assuming total dependency between measures (*r* = 1) as explained elsewhere [53]. Sensitivity analyses were also conducted by testing significance when removing one study at a time to check if findings were mostly driven by an individual study. Finally, sub-analyses were performed focusing solely on those studies with high and good quality according to the TESTEX scale, for those studies that applied a similar intervention in both SUP and UNSUP groups (e.g., both groups including exercise interventions targeting the same muscle groups and with similar characteristics) and for those studies in which participants performed more than two-thirds of the sessions in the assigned condition *(*i.e., the SUP group performed at least 66% of the sessions under supervision; Table 1, OSM Tables S3 and S4). Begg’s test was used to determine the presence of publication bias, and the I^2^ statistic was used to assess heterogeneity across studies. *I*^2^ values > 25%, 50%, and 75% were considered indicative of low, moderate, and high heterogeneity, respectively. The level of significance was set at 0.05. All statistical analyses were performed using the statistical software package Comprehensive Meta-analysis 2.0 (Biostat, Englewood, NJ, USA).

## Results

### Study Characteristics

**Fig. 1:**
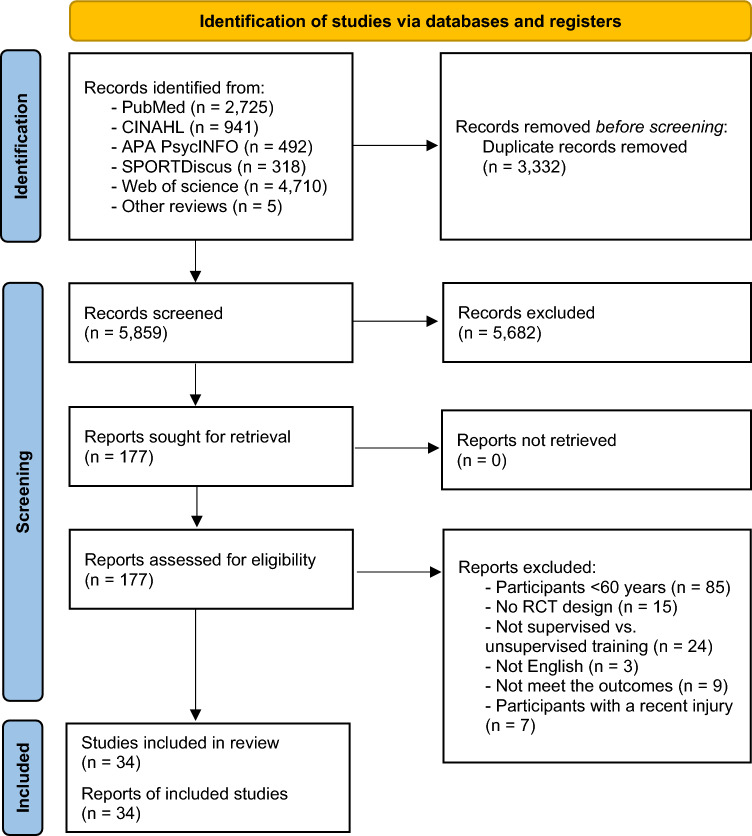
PRISMA 2020 flow diagram for new systematic reviews which included searches of databases, registers, and other sources. *RCT* randomized controlled trials

From the retrieved studies, 34 studies derived from 30 RCTs (*n* = 2830 participants) met all eligibility criteria and were included in the systematic review (Fig. 1). Seven studies analyzed the same sample from three RCTs [15, 18, 25, 26, 28, 29, 54], and they were only counted once for the final sample size. The characteristics of the included studies are summarized in Table 2.

**Table 2:** Characteristics of the included studies

Study	Sample demographics *(n*, sex, average age)	Exercise intervention						Attendance /	Endpoints	Main results
		Frequency	Intensity			Type of training	Duration			
Almeida et al. [24]	- SUP: *n* = 28 patients (22 female), 78 ± 4 years - UNSUP: *n* = 22 patients (20 female), 79 ± 5 years	SUP: 3 times/wk at a hospital center (50 min/session) UNSUP: Attended the center for 1 supervised session every other week, and they were encouraged to comply with sessions at home 3 times/wk (50 min/ session)	N/R			*Multicomponent training:* Strength and dual-task exercises, dynamic and static balancing and stretching	17 weeks	N/R	**- Safety:** Adverse events, falls **- Muscle strength:** STS **- Balance:** Berg balance scale, tandem-walk test, limits of stability test—forward **- Physical performance:** TUG, 400-m-walk time	SUP: ↑ STS _(weight-transfer time)_ ↑ Berg balance scale ↑ 400-m-walk time UNSUP: ↑ Tandem-walk test _(speed and end sway)_ SUP and UNSUP: ↑ Limits of stability test ↑ TUG - No differences in the remaining outcomes
Bieler et al. [18]	- SUP: *n* = 50 patients (34 female), 70 ± 5 years - UNSUP: *n* = 52 patients (36 female), 69 ± 6 years	SUP: 3 times/wk (2 group exercise sessions supervised and 1 unsupervised session) (60 min/session) UNSUP: 3 times/wk at the participant’s home (60 min/session)	SUP: 75% RM. Load increased from 20 to 10 RM (first 4 weeks). Load adjusted every second week or when the participant could perform > 10 reps UNSUP: 3–6 levels (strength) 1–3 sets of 10 reps. When participants no longer felt exhausted, they progressed to the next level			SUP: Strength training UNSUP*: Multicomponent training:* Strength training, hip ROM and stretching	17 weeks	Average attendance: SUP: 80% UNSUP: 90%	**- Muscle strength:** 30-s STS **- Physical performance**: TUG, TSC, 15-s MOS, 6MWT **- HRQoL**: SF-36	**SUP > UNSUP:** **↑ SF-36 subscales: role-physical, bodily pain and vitality** SUP and UNSUP: ↑ 30-s STS ↑ TUG - No differences in the remaining outcomes
Bieler et al. [26]	- SUP: *n* = 50 patients (34 female), 70 ± 5 years - UNSUP: *n* = 52 patients (36 female), 69 ± 6 years	SUP: 3 times/wk (2 group exercise sessions supervised and 1 unsupervised session) (60 min/session) UNSUP: 3 times/wk at the participant’s home (60 min/session)	SUP: 75%RM In cases of pain > 5 on a numeric pain rating scale (range 0–10) adjustments were made UNSUP: N/R			SUP: Strength training UNSUP*: Multicomponent training:* Strength training, hip ROM and stretching	17 weeks	Average attendance: SUP: 80% UNSUP: 90%	**- Muscle strength:** Leg extensor power, thigh muscle torque (knee extensors and flexors) and hip muscle torque (external and internal rotation, flexors, abductors, adductors) **- Physical performance**: Hip ROM (flexion, external and internal rotation)	SUP and UNSUP: ↑ Hip ROM: external and internal rotation - No differences in the remaining outcomes
Bieler et al. [25]	- SUP: *n* = 15 patients (11 female), 67 ± 4 years - UNSUP: *n* = 15 patients (13 female), 68 ± 5 years	SUP: 3 times/wk (2 group exercise sessions supervised and 1 unsupervised session) (60 min/session) UNSUP: 3 times/wk at the participant’s home (60 min/session)	SUP: 75%RM In cases of pain > 5 on a numeric pain rating scale (range 0–10) adjustments were made UNSUP: N/R			SUP: Strength training UNSUP*: Multicomponent training:* Strength training, hip ROM and stretching	17 weeks	N/R	**- Muscle strength:** Leg extensor power, maximal isometric knee strength, 30-s STS **- Physical performance**: TSC, 6MWT **- Body composition**: Quadriceps cross-sectional area	SUP: ↑ Leg power _EXT_ ↑ Maximal isometric knee strength ↑ 30-s STS ↑ TSC **↑ Quadriceps cross-sectional area (SUP > UNSUP)** - No differences in the remaining outcomes
Bittar et al. [27]	- SUP: *n* = 16 patients (16 female), 66 ± 7 years - UNSUP: *n* = 18 patients (18 female), 68 ± 6 years	SUP and UNSUP: 2 times/wk (60 min/ session)	N/R			*Multicomponent training:* Strength, stretching and impact exercises	52 weeks	Average attendance: SUP: N/R UNSUP: 62%	**- Body composition:** Total fat, FMI, lean arms, lean legs, ALMI, weight, android fat, gynoid fat, total lean, android/gynoid ratio	SUP: ↑ Lean arms and legs ↑ Total lean ↑ ALMI - No differences in the remaining outcomes
Boshuizen et al. [49]	- SUP: *n* = 16 patients (16 female), 80 ± 7 years - UNSUP: *n* = 16 patients (14 female), 79 ± 7 years	SUP: 3 times/wk (2 exercise sessions supervised and 1 unsupervised home session) (60 min/ session) UNSUP: 3 times/wk (1 exercise session supervised and 2 unsupervised home sessions) (60 min/ session)	N/R			Strength training	10 weeks	Average attendance: SUP: 79% UNSUP: 72%	**- Muscle strength:** Maximal isometric knee strength **- Balance:** Balance test **- Physical performance**: TUG, 20-m walking test, box-stepping test	SUP: ↑ Maximal isometric knee strength ↑ TUG ↑ 20-m walking test UNSUP: ↑ Maximal isometric knee strength (for participants with low initial torque) - No differences in the remaining outcomes
Brown et al. [16]	- SUP: *n* = 48 patients (28 female), 83 ± 4 years - UNSUP: *n* = 36 patients (22 female), 83 ± 4 years	SUP and UNSUP: 3 times/wk	Low intensity			*Multicomponent training:* Strength, balance, stretching, body handling skills, speed of reaction, and coordination exercises	13 weeks	N/R	**- Muscle strength:** Knee extension and flexion strength, hip abduction and extension, ankle plantar flexion and dorsiflexion strength, handgrip strength **- Balance:** FRT, one leg stance, Berg balance scale, Romberg test, balance beam **- Physical performance**: Physical performance test, ROM (shoulder flexion, external rotation, forward bend, trunk rotation, straight leg raise, hip flexor tightness, hip internal rotation, knee flexion, and dorsiflexion), gait speed, fast walk, cadence, obstacle course	**SUP > UNSUP:** **↑ Knee strength **_**EXT (60°º/s) and FLEX (60°º/s)**_ **↑ Physical performance test** UNSUP: **↑** Gait velocity **↑** Fast walk **↑ Cadence (UNSUP > SUP)** SUP and UNSUP: **↑ One leg stance (SUP > UNSUP)** **↑ Berg balance scale (SUP > UNSUP)** **↑ Romberg test: full tandem (SUP > UNSUP)** ↑ ROM: forward bend, trunk rotation, straight leg raise, hip internal rotation, and dorsiflexion **↑ Obstacle course (SUP > UNSUP)** - No differences in the remaining outcomes
Cecchi et al. [32]	- SUP: *n* = 25 patients (17 female), 73 ± 6 years - UNSUP: *n* = 25 patients (15 female), 72 ± 5 years	SUP: 2 times/wk (60 min/session) UNSUP: ≥ 2 times/wk (≥ 60 min/session)	SUP: Progressed from low to low-moderate, as tolerated UNSUP: N/R			SUP*: Multicomponent training:* Strength, balance, aerobic and stretching (low impact) UNSUP: Aerobic training: walking	13 weeks	Average attendance: SUP: 88% UNSUP: 84%	**- Safety:** Adverse events **- Muscle strength:** Hip flexion, knee extension and flexion, shoulder abduction strength **- Physical performance:** SPS, ROM (shoulder abduction-elevation, hip flexion and knee flexion), trunk flexibility **- HRQoL**: SF-36	**SUP > UNSUP:** **↑ Strength: hip **_**FLEX**_**, knee **_**FLEX/EXT**_** and shoulder abduction** **↑ SPS** **- ↑ ROM: shoulder abduction-elevation, hip and knee **_**FLEX**_ - **↑ SF-36 subscale: vitality** - No differences in the remaining outcomes
Costa et al. [17]	- SUP: *n* = 14 patients (14 female), 69 ± 6 years - UNSUP: *n* = 11 patients (11 female), 69 ± 7 years	SUP: 3 times/wk (60 min/session) UNSUP: 1 supervised group session/wk in the same room and 2 individual sessions/wk at home (60 min/session)	Borg rating of compensations or fatigue using ankle cuff weights from 1 to 4 kg			*Multicomponent training:* Strength and balance	12 weeks	Average attendance: SUP: 76% UNSUP: 72%	**- Safety:** Adverse events, falls **- Muscle strength:** Knee and hip peak torque/BW extensors and flexors, 5-STS **- Physical performance:** 4-m walk, usual and maximum gait speed, MRTWork extensors and flexors, power extensors and flexors, usual stride length and width	SUP: ↑ Knee MRTWork _FLEX (180°/s)_ ↑ Knee Power _FLEX (180°/s)_ ↑ Hip peak torque/BW_EXT (60°/s)_ ↑ Hip MRTWork _FLEX (60°/s)_ ↑ Hip Power _EXT (60°/s)_ ↑ 5-STS ↑ 4-m walk **↑ Gait speed **_**(usual and maximum speed)**_ (**SUP > UNSUP)** ↑ **Usual stride length** UNSUP: ↑ **Knee peak torque/BW **_**EXT (60° and 180º/s)**_** (UNSUP > SUP)** ↑ Knee peak torque/BW _FLEX (180º/s)_ SUP and UNSUP: ↑ Knee MRTWork _EXT and FLEX (60°/s)_ ↑ Hip MRTWork _EXT (60° and 180º/s)_ ↑ Hip power _EXT (60 and 180°/s)_ - No differences in the remaining outcomes
Cyarto et al. [28]	- SUP: *n* = 81 patients (61 female), 78 ± 7 years - UNSUP: *n* = 38 patients (31 female), 81 ± 6 years	SUP and UNSUP: 2 times/wk (60 min/ session)	N/R			*Multicomponent training:* Strength, balance and stretching	20 weeks	Average attendance: SUP: 66% UNSUP: 63%	**- Balance:** One leg stance, ABC score, tandem stance **- Physical performance:** TUG	SUP: **↑ One leg stance (SUP > UNSUP)** ↑ Tandem stance ↑ TUG UNSUP: **↑ ABC score (UNSUP > SUP)** - No differences in the remaining outcomes
Cyarto et al. [29]	- SUP: *n* = 81 patients (61 female), 78 years - UNSUP: *n* = 38 patients (31 female), 81 years	SUP and UNSUP: 2 times/wk (60 min/ session)	N/R			*Multicomponent training:* Strength, balance and stretching	20 weeks	Average attendance: SUP: 66% UNSUP: 63%	**- Muscle strength:** STS, arm curl test **- Physical performance:** TUG, 2-min step test, sit & reach, back scratch	SUP: ↑ TUG **↑ Sit & reach (SUP > UNSUP)** UNSUP: ↑ Back scratch SUP and UNSUP: ↑ STS ↑ Arm curl - No differences in the remaining outcomes
Donat et al. [30]	- SUP: *n* = 17 patients (10 female), 81* (range 19) years - UNSUP: *n* = 15 patients (10 female), 79* (range 21) years	SUP and UNSUP: 3 times/wk (45–50 min/ session)	Duration and nº of reps increased as the subjects’ tolerance increased and as time progressed			*Multicomponent training:* Strength, balance and stretching	8 weeks	Average attendance: SUP: 71% UNSUP: 88%	**- Muscle strength:** Leg strength **- Balance:** One leg stance, Berg balance scale, tandem standing **- Physical performance:** TUG, sit & reach, position sense	SUP: ↑ Leg strength ↑ Position sense SUP and UNSUP: ↑ One leg stance ↑ Berg balance scale ↑ Tandem standing ↑ TUG ↑ Sit & reach - No differences in the remaining outcomes
Ebersbach et al. [31]	- SUP: *n* = 20 patients (13 female), 67 ± 4 years - UNSUP: *n* = 19 patients (12 female), 69 ± 8 years	SUP: 4 times/wk (60 min/ session) UNSUP: N/R	Work with at least ‘‘80% of their maximal energy’’ on every repetition			SUP: Stretching, whole-body movements and goal-directed activities of daily living using high amplitude UNSUP: Stretching, high-amplitude movements and workouts for muscular power and posture	4 weeks	Average attendance: SUP: 100% UNSUP: N/R	**- Physical performance:** TUG, timed 10 m walking **- HRQoL**: PDQ-39	**SUP > UNSUP:** **↑ TUG** **↑ Timed 10 m walking** - No differences in the remaining outcome
Gardner et al. [33]	- SUP: *n* = 40 patients (18 female), 66 ± 12 years - UNSUP: *n* = 40 patients (18 female), 65 ± 11 years	SUP and UNSUP: 3 times/wk Session length: SUP: 15–40 min/session UNSUP: 20–45 min/ session	SUP: Lower-intensity program UNSUP: N/R			Aerobic training (walking)	12 weeks	Average attendance: SUP: 85% UNSUP: 83%	**- Safety:** Adverse events **- Physical performance:** VO_2peak_, walking economy test, gait analysis, average cadence **- HRQoL**: SF-36 subscale (physical function)	SUP: **↑ Walking economy (SUP > UNSUP)** UNSUP: ↑ Gait analysis: maximal cadences for 20, 30, and 60 min **(UNSUP > SUP for 30 min)** **↑ Average cadence (UNSUP > SUP)** SUP and UNSUP: ↑ SF-36 subscale: physical function - No differences in the remaining outcomes
Gardner et al. [34]	- SUP: *n* = 60 patients (29 female), 65 ± 11 years - UNSUP: *n* = 60 patients (31 female), 67 ± 10 years	SUP and UNSUP: 3 times/wk Session length: SUP: 15–40 min/session UNSUP: 20–45 min/ session	N/R			Aerobic training (walking)	12 weeks	Average attendance: SUP: 82% UNSUP: 81%	**- Safety:** Adverse events **- Physical performance**: 6MWT, walking economy test, gait analysis, average cadence **- HRQoL**: SF-36 subscale (physical function)	SUP: ↑ Walking economy UNSUP: ↑ Gait analysis: maximal cadences for 5, 20, 30, and 60 min ↑ Average cadence SUP and UNSUP: **↑ 6MWT (UNSUP > SUP)** ↑ Gait analysis: maximal cadences for 5 Data not shown for SF-36 subscale (physical function)
Hinman et al. [35]	- SUP: *n* = 28 patients (14 female), 73 years - UNSUP: *n* = 30 patients (23 female), 73 years	SUP and UNSUP: 3 times/wk (20 min/ session)	N/R			Balance training	4 weeks	Average attendance: SUP: 97% UNSUP: 92%	**- Balance:** Berg balance scale **- Physical performance**: Timed 50-foot walk test	SUP and UNSUP: ↑ Berg balance scale - No differences in the remaining outcomes
Horton et al. [36]	- SUP: *n* = 142 patients (66 female), 67 ± 8 years - UNSUP: *n* = 145 patients (64 female), 68 ± 9 years	SUP: Daily training walks (30 min/session); 2 exercise sessions hospital-based supervised (60 min/ session) UNSUP: Daily training walks (30 min/session); Strength: 3 times/wk	*Aerobic:* Walking at the correct speed (intensity) using Borg breathlessness scores *Strength:* Increase the resistance once their Borg scores became easier (lower)			*Multicomponent training:* Strength and aerobic (walking)	7 weeks	N/R	**- Safety:** Adverse events **- Physical performance**: Endurance and incremental shuttle walk test	SUP and UNSUP: **↑ Endurance shuttle walk test (SUP > UNSUP)** ↑ Incremental shuttle walk test - No differences in the remaining outcomes
Iliffe et al. [37]	- SUP: *n* = 387 patients (239 female), 73 ± 6 years - UNSUP: *n* = 410 patients (260 female), 73 ± 6 years	SUP: 1 exercise session center-based supervised (60 min/session), 2 home exercise sessions (30 min/session) and walking ≥ 2 times/wk (≤ 30 min/session) UNSUP: Strength and balance: ≥ 3 times/wk (30 min/session) and walking ≥ 2 times/wk (≤ 30 min/ session)	SUP: More intense than UNSUP UNSUP: Moderate intensity		*Multicomponent training*: Strength, balance and aerobic (walking)		24 weeks	Participants who had attendance rates of ≥ 75% of expected total sessions: SUP: 17% UNSUP: 25%	**- Safety:** Adverse events, falls **- Muscle strength:** 30-s STS **- Balance:** Modified clinical Romberg static balance test, eyes open and closed, FRT **- Physical performance**: TUG **- HRQoL**: OPQoL, SF-12, EQ-5D	- There were no differences in any of the results
Kakkos et al. [48]	- SUP: *n* = 12 patients (1 female), 69* (IQR 12) years - UNSUP: *n* = 9 patients (1 female), 66* (IQR 11) years	SUP: 3 times/wk (60 min/ session) UNSUP: N/R	N/R	Aerobic training (walking)			26 weeks	Average attendance: SUP: 60% UNSUP: N/R	**- Physical performance**: Walking distance, stair climbing **- HRQoL**: SF-36	SUP: **↑ Walking distance (SUP > UNSUP)** - No differences in the remaining outcomes
Karahan et al. [50]	- SUP: *n* = 48 patients (21 female), 71 ± 6 years - UNSUP: *n* = 42 patients (18 female), 72 ± 5 years	SUP and UNSUP: 5 times/wk (30 min/ session)	11–13 RPE ("light performance" to "somewhat hard performance")	SUP: *Aerobic training*: Exergames UNSUP: *Multicomponent training*: Strength, balance and stretching			6 weeks	Average attendance: SUP: 100% UNSUP: 100%	**- Balance:** Berg balance scale **- Physical performance**: TUG **- HRQoL**: SF-36	SUP: ↑ TUG ↑ SF-36 subscales: physical functioning, social role functioning, physical role restriction, general health perceptions, and physical component scores SUP and UNSUP: **↑ Berg balance scale (SUP > UNSUP)**
Lacroix et al. [55]	- SUP: *n* = 22 patients (14 female), 73 ± 4 years - UNSUP: *n* = 22 patients (14 female), 73 ± 4 years	SUP: 2 times/wk supervised at a local gym and 1/wk unsupervised at home (45 min/session) UNSUP: 3 times/wk (45 min/session)	12–16 RPE (‘somewhat hard’ to ‘hard’)	*Multicomponent training:* Strength and balance			12 weeks	Average attendance: SUP: 92% UNSUP: 97%	**- Safety:** Adverse events **- Muscle strength:** STS **- Balance:** Stride length, FRT, modified Romberg test, posturomed ML, PRT score **- Physical performance**: TUG, stair ascent and descent test **- Body composition:** BMI, body mass, lean tissue mass of the legs, total body water, total skeletal muscle mass	SUP: ↑ Romberg test (s) ↑ TUG UNSUP: ↑ Posturomed ML ↓ Lean tissue mass of the legs SUP and UNSUP: **↑ STS (SUP > UNSUP)** ↑ FRT ↑ PRT score ↑ Stair ascent and descent test - No differences in the remaining outcomes
Lindemann et al. [38]	- SUP: *n* = 12 patients (9 female), 69* (range 60–78) years - UNSUP: *n* = 12 patients (9 female), 72* (range 60–82) years	SUP and UNSUP: 2 times/wk (20 min/ session)	N/R	Balance training			8 weeks	Average attendance: SUP: N/R UNSUP: 98%	**- Balance:** FRT, summary balance score, maximum step length test **- Physical performance**: Maximum gait speed	SUP: ↑ Summary balance score ↑ Gait speed - No differences in the remaining outcomes
Mickle et al. [39]	- SUP: *n* = 43 patients (35 female), 71 ± 6 years - UNSUP: *n* = 42 patients (31 female), 70 ± 6 years	SUP and UNSUP: 3 times/wk (45 min/ session)	SUP: Increasing either the strength of the resistance bands or the nº of reps UNSUP: Exercises that had little or no resistance and did not progress in amount of resistance	Strength training			12 weeks	Average attendance: SUP: 89% UNSUP: 83%	**- Safety:** Adverse events **- Muscle strength:** Toe strength **- Balance:** One leg stance (with eyes open and closed) **- HRQoL:** Foot QoL (foot pain, foot function, shoes, general foot health, general health, physical activity, social capacity, vigour)	SUP: ↑ Toe strength ↑ One leg stance with eyes open ↑ General foot health SUP and UNSUP: ↑ One leg stance with eyes closed - No differences in the remaining outcomes
Morrison et al. [40]	- SUP: *n* = 32 patients (15 female), 68 ± 5 years - UNSUP: *n* = 14 patients (7 female), 66 ± 6 years	SUP and UNSUP: 3 times/wk (40 min/ session)	N/R	Balance training			12 weeks	SUP: N/R UNSUP: > 50% failing to appropriately complete the training or all sessions	**- Muscle strength:** Knee extension and flexion strength **- Balance:** Postural sway	SUP and UNSUP: **↑** Postural sway - No differences in the remaining outcomes
Nai-Hsin et al. [41]	- SUP: *n* = 74 patients (40 female), 76 ± 6 years - UNSUP: *n* = 72 patients (42 female), 77 ± 7 years	SUP: 3 times/wk (90 min/session) UNSUP: ≥ 3 times/wk (40–45 min/session)	SUP: 70–85% of the predicted maximum heart rate (220—age) or 13 RPE UNSUP: N/R	*Multicomponent training:* Strength and aerobic			13 weeks	Average attendance: SUP: 74% UNSUP: N/R	**- Safety:** Adverse events **- Muscle strength:** Knee extension and flexion strength, 3-STS, 1-RM leg press strength, handgrip strength, elbow flexion **- Balance:** One leg stance **- Physical performance**: TUG, maximum gait speed, 6MWT **- Body composition:** Body fat, lean mass, circumferences of the waist, hip, upper arms, thighs and legs	SUP: ↑ 3-STS **↑ Leg press strength (SUP > UNSUP)** ↑ **TUG (SUP > UNSUP)** ↑ Leg circumference ↑** Upper arm circumference (SUP > UNSUP)** UNSUP: ↑ Handgrip strength ↑ Elbow strength _FLEX_ ↓ Thigh circumference SUP and UNSUP: ↑ Knee strength _EXT_ ↑ Knee strength _FLEX_ ↑ Gait speed **↑** % fat - No differences in the remaining outcomes
Opdenacker et al. [54]	- SUP: *n* = 60 patients (30 female), 70 ± 4 years - UNSUP: *n* = 60 patients (30 female), 66 ± 4 years	SUP: 3 times/wk (60– 90 min/session) UNSUP: N/R	SUP: 70–80% of the individual heart rate reserve UNSUP: N/R	*Multicomponent training:* Strength, balance, aerobic and stretching			44 weeks	Participants who attended to their program: SUP: 80% UNSUP: 78%	**- Muscle strength:** Static strength, dynamic strength, STS, arm curl **- Physical performance**: VO_2peak_, total work, vertical jump **- Body composition:** BMI, % body fat, waist circumference	**SUP > UNSUP:** **↑ Static strength** **↑ Dynamic strength** **↑ VO**_**2peak**_ **↑ Total work** - No differences in the remaining outcomes
Perez-Dominguez et al. [43]	- SUP: *n* = 36 patients (12 female), 67 ± 13 years - UNSUP: *n* = 34 patients (12 female), 67 ± 16 years	SUP and UNSUP: 3 times/wk (60 min/ session)	12 RPE (“somehow hard” effort)	*Multicomponent training:* Strength, balance, aerobic and stretching			16 weeks	N/R	**- Muscle strength:** 10- and 60-STS, handgrip strength **- Balance:** One leg stance **- Physical performance**: TUG, gait speed, 6MWT, SPPB, one leg heel rise **- HRQoL**: SF-36	SUP: ↑ Handgrip strength ↑ TUG UNSUP: ↑ One leg stance SUP and UNSUP: ↑ 10-STS ↑ Gait speed ↑ 6MWT ↑ SPPB ↑ One leg heel rise ↑ SF-36: all subscales except social functioning - No differences in the remaining outcomes
Sandberg et al. [56]	- SUP: *n* = 54 patients (23 female), 72 ± 8 years - UNSUP: *n* = 56 patients (21 female), 72 ± 7 years	SUP and UNSUP: 3 times/wk (50 min/ session)	13–15 RPE	*Multicomponent training:* Strength and aerobic			26 weeks	Participants achieved ≥ 80% of the exercise sessions at prescribed intensity (adherence): SUP: 26% UNSUP: 24% Attendance at ≥ 20 to < 80% of exercise sessions regardless of intensity (attendance): SUP: 48% UNSUP: 71%	**- Muscle strength:** STS **- Physical performance**: 6MWT, one leg heel rise	UNSUP: ↑ 6MWT SUP and UNSUP: ↑ STS ↑ One leg heel rise
Sian et al. [47]	- SUP: *n* = 10 patients (5 female), 70 ± 5 years - UNSUP: *n* = 10 patients (7 female), 71 ± 4 years	SUP and UNSUP: 3 times/wk (15 min/ session)	85% HRmax	*Multicomponent training:* Strength and aerobic (HIIT)			4 weeks	Average attendance and adherence: SUP: 100% UNSUP: 100%	**- Safety:** Adverse events **- Physical performance:** VO_2peak_ **- Body composition**: Fat mass and lean mass	SUP and UNSUP: ↑ VO_2peak_ ↑ Fat mass - No differences in the remaining outcomes
Tsekoura et al. [44]	- SUP: *n* = 18 patients (16 female), 75 ± 6 years - UNSUP: *n* = 18 patients (15 female), 71 ± 7 years	SUP and UNSUP: training program 2 times/wk (50–70 min/session) and walk for 100 min/wk (≤ 30–35 min/ session; 3 times/wk)	Based on Borg’s RPE scale	*Multicomponent training:* Strength and aerobic			12 weeks	Average attendance: SUP: 92% UNSUP: 88%	**- Safety:** Adverse events **- Muscle strength:** Right and left knee flexion and extension strength (90º and 180º), STS, handgrip strength **- Physical performance**: TUG, 4 m test, gait speed **- Body composition**: BMI, SMMI, fat free mass, calf circumference **- HRQoL**: SarQoL	SUP: ↑ Right knee _EXT (90º and 180º/s)_ ↑ Right knee _FLEX (90º º/s)_ ↑ Left knee _EXT (90º/s)_ **↑ Left knee **_**FLEX (90º/s).**_** (SUP > UNSUP)** **↑ Handgrip strength. (SUP > UNSUP)** **↑ SMMI. (SUP > UNSUP)** **↑ Calf circumference. (SUP > UNSUP)** SUP and UNSUP: **↑ Right knee **_**FLEX (180º/s).**_** (SUP > UNSUP)** ↑ Left knee _FLEX (180º/s)_ **↑ STS. (SUP > UNSUP)** **↑ TUG** **↑ 4 m test. (SUP > UNSUP)** **↑ Gait speed. (SUP > UNSUP)** - No differences in the remaining outcomes
Van Roie et al. [15]	- SUP: *n* = 60 patients (30 female), 67 ± 5 years - UNSUP: *n* = 60 patients (30 female), 66 ± 4 years	SUP: 3 times/wk (60–90 min/session) UNSUP: N/R	SUP: 70–80% of the individual heart rate reserve UNSUP: N/R	*Multicomponent training:* Strength, balance, aerobic and stretching			44 weeks	Participants who attended to their program: SUP: 80% UNSUP: 78%	**- Muscle strength:** Static strength, dynamic strength, STS, arm curl **- Physical performance**: VO_2peak_, total work, vertical jump **- Body composition:** BMI, % body fat, waist: hip ratio, waist circumference, hip circumference	**SUP > UNSUP:** **↑ Static strength** **↑ Dynamic strength** **↑ VO**_**2peak**_ **↑ Total work** **↑ Hip circumference** - No differences in the remaining outcomes
Watanabe et al. [45]	- SUP: *n* = 243 patients (139 female), 74 ± 5 years - UNSUP: *n* = 274 patients (161 female), 74 ± 6 years	SUP and UNSUP: weekly 90-min sessions	Low intensity	Strength training			12 weeks	N/R	**- Muscle strength:** Knee extension strength, 5-STS, 30-s STS, handgrip strength **- Balance:** FRT **- Physical performance**: TUG, usual and maximum gait speed, chair stepping, vertical jump index **- Body composition**: Anterior thigh muscle thickness	SUP: **↑ TUG (SUP > UNSUP)** **↑ Maximum gait speed (SUP > UNSUP)** SUP and UNSUP: **↑ Knee strength **_**EXT**_** (SUP > UNSUP)** ↑ 5-STS ↑ 30-s STS ↑ FRT ↑ Usual gait speed ↑ Chair stepping ↑ Vertical jump index ↑ Anterior thigh muscle thickness - No differences in the remaining outcomes
Watson et al. [14]	- SUP: *n* = 12 patients (12 female), 65 ± 4 years - UNSUP: *n* = 16 patients (16 female), 67 ± 5 years	SUP and UNSUP: 2 times/wk (30 min/ session)	SUP: > 80–85% RM UNSUP: Low-intensity (< 60% RM). Strength exercises progressed from body weight to a maximum of 3 kg hand weights	Strength training			35 weeks	Average attendance: SUP: 87% UNSUP: 93%	**- Safety:** Adverse events **- Muscle strength:** 5-STS, back extensor strength **- Balance:** FRT **- Physical performance**: TUG **- Body composition:** FN and LS BMD, BMI, weight, lean and fat mass	**SUP > UNSUP:** **↑ 5-STS** **↑ Back extensor strength** **↑ FRT** **↑ FN and LS BMD** SUP: **↑** Fat mass - No differences in the remaining outcomes
Wu et al. [46]	- SUP: *n* = 20 patients (16 female), 74 ± 7 years - UNSUP: *n* = 22 patients (19 female), 76 ± 6 years	SUP and UNSUP: 3 times/wk (60 min/ session)	N/R	Balance training (Tai Chi)			15 weeks	Average attendance: SUP: 71% UNSUP: 38%	**- Safety:** Falls **- Balance:** One leg stance, body sway during quiet stance **- Physical performance**: TUG **- HRQoL**: SF-36	SUP: **↑** One leg stance ↑ Body sway with eyes open **↑** SF-36 subscales: social function, physical health, bodily pain, and physical function ↑ SF-36 subscales: mental health, and vitality UNSUP: ↑ SF-36 subscale: role physical - No differences in the remaining outcomes

*ABC* activities-specific balance confidence scale, *ALMI* appendicular lean mass index, *BMD* bone mineral density, *BMI* body mass index, *BW* body weight, *EQ-5D* European Quality of Life-5 Dimensions, *EXT* extension, *FLEX* flexion, *FN* femoral neck, *FRT* functional reach test, *HIIT* high-intensity interval training, *HRQoL* health-related quality of life, *IQR* interquartile range, *LS* lumbar spine, *ML* mediolateral, *MOS* marching on the spot test, *MRTWork* maximal repetition total work, *N/R* not reported, *OPQoL* Older people’s quality of life questionnaire, *PDQ* Parkinson’s disease questionnaire, *PRT* push and release test, *QoL* quality of life, *RM* repetition maximum, *ROM* range of motion, *RPE* rate of perceived exertion, *SarQoL* Sarcopenia quality of life questionnaire, *SF-12* 12-item short-form health survey, *SF-36* 36-item short form health survey, *SMMI* skeletal muscle mass index, *SPPB* Short physical performance battery, *SPS* Summary of performance score, *STS* sit-to-stand test, *SUP* supervised exercise group, *TSC* timed stair climbing test, *TUG* timed-up-and-go test, *UNSUP* unsupervised exercise group, *6MWT* 6-min walk test

^*^Data are shown as median

↑ Significant improvement in the outcome

↓ Significant worsening in the outcome

Participants’ average age ranged from 65 to 83 years (weighted average 72). Participants’ characteristics were highly heterogeneous, including different types of populations such as participants with hip osteoarthritis [18, 25, 26], sarcopenia [44], pre-frailty and frailty [16, 17, 41, 49], osteopenia and osteoporosis [14], Parkinson’s disease [31], intermittent claudication [33, 48], chronic obstructive pulmonary disease [36], previous falls [24, 46], type 2 diabetes [40], peripheral artery disease [34], postmenopausal women [27], sedentary individuals [15, 32, 54], individuals undergoing hemodialysis [43], independent individuals who resided in retirement village residents [28, 29] or in a nursing home [30], and community-dwelling older adults [35, 37–39, 45, 47, 55].

SUP and UNSUP included two to five training sessions per week (~ 15–90 min per session) and two to six training sessions per week (~ 15–60 min per session), respectively, and lasted between 4 and 52 weeks for both interventions. Only 18 studies (53%) included the same type of exercise intervention for both groups (in some studies the SUP and UNSUP groups performed exactly the same exercise program so only differed in the amount of guidance received while in other studies the exercises were similar but adapted to be performed at home) including strength training in two studies [45, 49], balance training in four studies [35, 38, 40, 46], aerobic training in one study [48] or multicomponent training (i.e., mainly strength, balance, aerobic, and/or stretching exercises combined in the same session) in the remaining 11 studies [24, 27–30, 36, 43, 44, 47, 55, 56]. For the other 16 studies, the exercise intervention performed between SUP and UNSUP groups was different. SUP was not fully supervised in eight out of 34 studies [18, 25, 26, 36, 37, 45, 49, 55]. Most studies provided between one and eight supervised sessions as instruction or as follow-up during the intervention to ensure that the exercise program was properly implemented [15, 18, 25, 26, 28–31, 33, 35, 38, 40, 41, 43, 47, 50, 54, 56], while others provided telephone follow-up [27–29, 38]. Additionally, in some studies, the UNSUP group also included from three up to 12 supervised training sessions [16, 17, 24, 44, 49].

### Quality Assessment and Publication Bias

The quality of the included studies was overall good (mean TESTEX score of 10, range 4–14; Table 3). Three (9%) of the studies showed low methodological quality, 17 (50%) were of good quality, and 14 (41%) were deemed to be of high quality. Most studies did not specify allocation concealment (76% of the studies) or did not report adverse events (56% of the studies). Also, only 41% of the studies had a completion rate of at least 85% and only 41% reported details on assessors’ blinding.

**Table 3:**
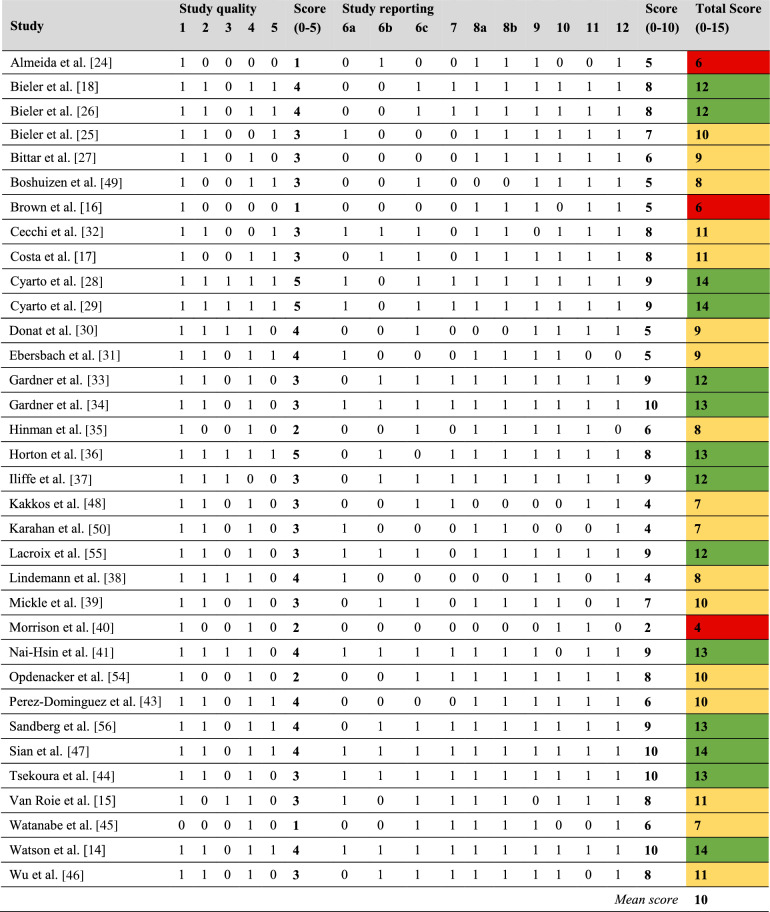
Quality of the included studies using the Tool for the assEssment of Study qualiTy and reporting in Exercise (TESTEX) scale

**Study quality:** 1 = Eligibility criteria specified; 2 = Randomization specified; 3 = Allocation concealment; 4 = Groups similar at baseline; 5 = Blinding of assessor (for at least one key outcome). **Study reporting:** 6 = Outcome measures assessed in 85% of participants (6a = 1 point if completion rate is [85%; 6b = 1 point if adverse events are reported; 6c = 1 point if exercise attendance is reported); 7 = Intention-to-treat analysis; 8 = Between-group statistical comparisons reported (8a = 1 point if between-group statistical comparisons are reported for the primary outcome measure of interest; 8b = 1 point if between-group statistical comparisons are reported for at least one secondary outcome measure); 9 = Point measures and measures of variability for all reported outcome measures; 10 = Activity monitoring in control groups; 11 = Relative exercise intensity remained constant; 12 = Exercise volume and energy expenditure. The studies were classified according to their total TESTEX score as ‘high quality’ (≥ 12 points, green color), ‘good quality’ (7 to 11 points, yellow color), or ‘low quality’ (≤ 6 points, red color)

### Endpoints

#### Main Analyses

##### Safety

Thirteen of the 34 included studies registered the incidence of adverse events [14, 17, 24, 32–34, 36, 37, 39, 41, 44, 47, 55] and four registered the number of falls [17, 24, 37, 46] during the study period, with none of them reporting significant differences between groups.

##### Attendance and Adherence Rates

Twenty-eight studies involving 25 RCTs reported attendance rates. Twenty-one studies computed attendance as the proportion of sessions completed from those initially prescribed for SUP [14, 17, 18, 26, 28–35, 39, 41, 44, 46–50, 55], reporting a weighted average attendance of 81% (range 60–100%). On the other hand, 20 studies registered the attendance rates for UNSUP, reporting a weighted attendance of 81% (range 38–100%) [14, 17, 18, 26–30, 32–35, 38, 39, 44, 46, 47, 49, 50, 55]. Two studies considered whether participants complied with variables such as intensity, duration, and prescribed exercises in addition to attendance at sessions (adherence) [47, 56]. Sandberg et al. [56] reported that 24% and 26% of the participants in SUP and UNSUP groups, respectively, were classified as fully adherent (i.e.*,* achieved ≥ 80% of the exercise sessions at prescribed intensity), while 71% and 48%, respectively, were partially adherent (i.e.*,* attendance at ≥ 20% to < 80% of exercise sessions regardless of intensity). Iliffe et al. [37] reported that 17% and 25% of the participants in the SUP and UNSUP groups, respectively, completed ≥ 75% of the prescribed sessions. Morrison et al. [40] reported that > 50% of subjects in the UNSUP group did not adequately complete the training sessions. Opdenacker et al. [54] and Van Roie et al. [15] reported that 80% and 78% of participants adhered to the exercise program (i.e., completed ≥ 80% of their program) in the SUP and UNSUP groups, respectively. The remaining six studies [16, 24, 25, 36, 43, 45] did not provide data on compliance rates for any of the groups.

##### Muscle Strength

Seventeen studies assessed different strength-related measures [14–17, 25, 26, 29, 30, 32, 39–41, 43–45, 49, 54], of which 13 could be meta-analyzed (OSM Figs. S1–S4). SUP induced significantly superior benefits to UNSUP on knee extension strength, which was confirmed in sensitivity analyses (Table 4). Furthermore, significant benefits of SUP were found for the sit-to-stand test (STS), but this was not significant in sensitivity analyses. No significant differences between SUP and UNSUP groups were found for handgrip strength.

**Table 4:** Summary of pooled results

Outcome	Studies (participants)	Effect estimate (95%CI)	*p*-value	*I*^2^	Begg’s *p*-value	Significant in sensitivity analyses	Quality (mean TESTEX score, range)
Muscle strength							
Knee extension strength (SMD)	10 (*n* = 1,126)	0.18 (0.07, 0.30)	**0.002**	0%	0.037	Yes	9 (4–13)
Knee flexion strength (SMD)	6 (*n* = 439)	0.23 (– 0.04, 0.50)	0.089	41.7%	0.226	No	10 (4–13)
STS (SMD)	12 (*n* = 1,745)	0.25 (0.00, 0.50)	**0.050**	80.9%	0.008	No	12 (7–14)
Handgrip (SMD)	4 (*n* = 755)	0.13 (– 0.18, 0.45)	0.404	65.3%	0.154	No	11 (7–13)
Balance							
Functional reach test (SMD)	6 (*n* = 1,165)	0.45 (– 0.03, 0.93)	0.064	89.9%	0.130	No	10 (6–14)
One leg stance (SMD)	5 (*n* = 438)	0.19 (– 0.21, 0.59)	0.351	74.7%	0.500	No	10 (6–14)
Balance scales (SMD)	5 (*n* = 375)	0.28 (– 0.32, 0.88)	0.356	86.7%	0.403	No	8 (6–14)
Tandem stance with eyes closed (SMD)	4 (*n* = 202)	0.10 (– 0.42, 0.61)	0.715	62.3%	0.148	No	8 (4–12)
Tandem stance with eyes open (SMD)	4 (*n* = 229)	– 0.01 (– 0.66, 0.65)	0.983	79.1%	0.045	No	9 (4–14)
Physical performance							
TUG (SMD)	15 (*n* = 1,711)	0.21 (0.02, 0.40)	**0.035**	66.8%	0.138	No	11 (6–14)
Usual gait speed (SMD)	8 (*n* = 834)	0.29 (0.04, 0.55)	**0.026**	52.2%	0.054	No	9 (6–13)
Maximum gait speed (SMD)	6 (*n* = 841)	0.17 (– 0.03, 0.38)	0.100	33.6%	0.500	No	9 (6–13)
6MWT (m)	5 (*n* = 504)	1.8 (– 15.5, 19.1)	0.838	34.1%	0.500	No	12 (10–13)
VO_2peak_ (ml/kg/min)	3 (*n* = 202)	0.32 (– 0.83, 1.47)	0.583	15.5%	0.500	No	12 (10–14)
Body composition							
BMI (kg/m^2^)	3 (*n* = 302)	– 0.03 (– 0.73, 0.67)	0.933	0%	0.148	No	12 (10–13)
Body mass (kg)	3 (*n* = 208)	– 0.36 (– 0.78, 0.06)	0.092	0%	0.500	No	12 (9–14)
Body fat (SMD)	5 (*n* = 348)	0.13 (– 0.13, 0.39)	0.314	21.7%	0.231	No	12 (9–14)
Lean mass (kg)	5 (*n* = 118)	1.05 (0.76, 1.34)	** < 0.001**	0%	0.367	No	12 (9–14)
HRQoL (SMD)	9 (*n* = 953)	0.21 (0.02, 0.40)	**0.035**	63.1%	0.059	No	10 (7–13)

*BMI* body mass index, *HRQoL* Health-related quality of life, *STS* Sit-to-stand test, *TESTEX* Tool for the assEssment of Study qualiTy and reporting in Exercise, *TUG* Timed-up-and-go test, *VO*_*2peak*_ maximal oxygen uptake, *6MWT* 6-min walk test

Results are shown as standardized mean difference (SMD) or absolute mean difference along with 95% confidence intervals (CI). A higher TESTEX score indicates better quality. Significant p-values for the effect estimates are in bold font

##### Balance

Seventeen studies evaluated balance-related endpoints [14, 16, 24, 28, 30, 35, 37–41, 43, 45, 46, 49, 50, 55], of which 14 could be included in the analyses (OSM Figs. S5–S9). Significantly superior benefits were found for SUP when pooling the four studies that assessed the Berg balance scale, but significance was not confirmed in sensitivity analyses. A non-significant trend towards beneficial effects of SUP was observed for the functional reach test (FRT), although when removing the study by Watson et al. [14], the result was far from significant (Table 4). No significant differences between interventions were found for one leg stance or tandem stance with eyes closed or open.

##### Physical Performance

Thirty-one studies measured endpoints related to physical performance (i.e., timed-up-and-go test (TUG), usual and maximum gait speed, 6-min walk test, and maximal oxygen uptake) [14–18, 24–26, 28–38, 41, 43–50, 54–56] and 25 of them could be meta-analyzed (OSM Figs. S10–S14). SUP induced significantly superior benefits to UNSUP on TUG and usual gait speed, although these results were not significant in sensitivity analyses (Table 4). On the other hand, no significant benefits were observed for maximum gait speed, 6-min walk test, or maximal oxygen uptake.

##### Body Composition

Ten studies assessed different markers of body composition [14, 15, 25, 27, 41, 44, 45, 47, 54, 55] and seven could be included in the analyses (OSM Figs. S15–S18). No significant differences were found between SUP and UNSUP for body mass index, body mass, or body fat (Table 4). Nevertheless, significantly superior benefits of SUP were found for lean mass, although these differences became non-significant in sensitivity analyses when removing the study by Watson et al. [14]. Some studies analyzed other body composition variables such as bone mineral density [14] or body circumferences [15, 41, 44, 54], but they could not be meta-analyzed (Table 2).

##### Health-Related Quality of Life

Twelve studies assessed HRQoL [18, 31–34, 37, 39, 43, 44, 46, 48, 50], of which nine could be included in the analyses (OSM Fig. S19). Significant benefits of SUP over UNSUP were found for HRQoL, but sensitivity analysis showed that the removal of almost each individual study (except for Kakkos et al. [48], Illife et al. [37], or Pérez-Dominguez et al. [43]) made the results non-significant.

The results obtained in the main analyses remained essentially the same after removing the low-quality studies except for usual gait speed, which became non-significant, and the functional reach test, which became significant (see OSM Table S4).

#### Sub-Analyses of Confounding Factors

##### Muscle Strength

Beneficial effects of SUP on knee extension strength were observed when separately analyzing those studies that applied a similar intervention [30, 40, 44, 45, 49] and in the nine studies [16, 17, 26, 30, 40, 41, 44, 49, 54] in which participants performed ≥ 66% of the sessions in the assigned condition in both groups (OSM Table S3). Sub-analysis also confirmed significantly superior benefits of SUP in those studies [29, 43–45, 55, 56] where participants performed ≥ 66% of the sessions in the assigned condition (Table 1) for STS. No significant differences were found for handgrip strength in sub-analyses.

##### Balance

There were no significant benefits of SUP on the FRT when analyzing those studies [38, 45, 55] that applied a comparable intervention, although significant benefits were found in those studies in which participants performed ≥ 66% of the sessions in the assigned condition [14, 16, 38, 55] (Supplementary Table S3). No additional benefits were found when pooling the three studies that applied a similar training intervention in the SUP and UNSUP groups for one leg stance [28, 30, 43], balance scales [24, 28, 30], and tandem stance with eyes closed [30, 40, 55]. Participants in all studies completed ≥ 66% of the sessions in the allocated intervention for one leg stance [16, 28, 30, 41, 43], balance scales [16, 24, 28, 30, 50], and tandem stance with eyes closed [16, 30, 40, 55] and open [28, 30, 40, 49].

##### Physical Performance

A trend towards statistical significance was observed in those studies that applied a similar intervention in both SUP and UNSUP groups on TUG [24, 28, 30, 43–46, 49, 55] and usual gait speed [24, 43–45, 49], but not on maximum gait speed [35, 38, 45] (*p* = 0.274; OSM Table S3). Sub-analysis also confirmed significantly superior benefits of SUP in those studies in which participants performed ≥ 66% of the sessions in the assigned condition for TUG [14, 18, 24, 28, 30, 31, 41, 43, 44, 46, 49, 50, 55], and usual gait speed [16, 17, 24, 31, 43, 44, 49], but not for maximum gait speed [16, 17, 35, 38, 41], 6-min walk test [18, 34, 41, 43, 56], and maximal oxygen uptake [33, 47, 54].

##### Body Composition

Three studies performed a comparable training intervention in SUP and UNSUP for lean mass [27, 44, 47], and no significant benefits were found when pooling these studies. In all studies, participants performed ≥ 66% of the sessions in the assigned condition and significant benefits of SUP over UNSUP were found for lean mass (OSM Table S3).

##### Health-Related Quality of Life

Four studies [43, 44, 46, 48] applied a similar intervention in both SUP and UNSUP groups, and no differences were found when separately analyzing them. In seven [18, 31, 39, 43, 44, 46, 50] out of the nine studies participants performed ≥ 66% of the sessions in the assigned condition, and their separate analyses revealed significant benefits of SUP, whereas non-significant benefits were found for the two studies that did not meet this criterion (OSM Table S3).

## Discussion

The present systematic review and meta-analysis compared the safety, attendance/adherence rates, and effectiveness of SUP versus UNSUP on measures of physical function and well-being outcomes in older adults. The incidence of adverse events and falls as well as the attendance to the program (81%) were similar in the SUP and UNSUP groups. Compared to UNSUP, SUP provided significantly superior benefits in knee extension strength, STS, TUG, usual gait speed, lean mass, and HRQoL, but only knee extension strength was still significant after sensitivity analyses. No benefits were found for the remaining outcomes. These results highlight the potential additional benefits that SUP can provide over UNSUP in older adults. However, for those unable to perform SUP, UNSUP may represent a safe and cost-effective alternative to ensure physical exercise.

### Safety

We found that most of the included studies reported overall similar rates of adverse events and falls in SUP and UNSUP. For example, Almeida et al. [24], Costa et al. [17], and Lacroix et al. [56] performed a multicomponent exercise intervention (combining strength and aerobic training) during 12 weeks and reported no adverse events (e.g., falls, muscle soreness, or injuries) during the study for either group. Furthermore, one of the included studies with the longest duration (i.e., 35 weeks) did not register any adverse events as a result of the exercise programs [14]. Our results are in accord with a systematic review and meta-analysis analyzing the safety and effectiveness of long‑term (≥ 1 year) exercise interventions in older adults, which concluded that regardless of supervision or intervention structure (i.e., supervised group-based, unsupervised home-based, or a combination thereof), exercise reduces the number of falls and fall-associated injuries in this population [5]. However, it must be noted that the number of adverse events and falls reported in this study may be underestimated given that exercise dose variables are generally not equated between SUP and UNSUP groups (i.e., more difficult exercise selection, higher intensity, and volume for SUP).

### Attendance and Adherence to the Exercise Program

In the present study we observed attendance rates of 81% for both SUP and UNSUP groups. In this regard, Lacroix et al. [13] compared the effects of SUP versus UNSUP programs including resistance and balance exercises on different physical fitness measures in older adults, finding a lack of association between attendance rates and the total number of supervised sessions. However, it is worth noting that there are at least two factors that might bias attendance rates. Firstly, most of the studies reported attendance using diaries in the UNSUP group, so the data obtained may not be accurate. Secondly, the fact that 21 of the 34 studies involved some level of supervision in the UNSUP group may affect the attendance rates obtained.

Another relevant finding is that only two studies considered whether participants complied with the prescribed parameters (i.e.*,* intensity, duration, exercises) as well as their attendance to the training sessions (adherence). There are factors that can promote greater long-term adherence to the exercise program [57]. Although in the present study attendance rates were similar between groups, it is unclear whether this attendance rate could be maintained in the long term. One of the studies that showed the greatest benefits of SUP versus UNSUP lasted 35 weeks and had attendance rates over 85% in both groups [14]. Therefore, the limited duration of most interventions or the low attendance to the program in other studies may explain the lack of significant benefits observed in the remaining outcomes. In this sense, previous research has shown that people may be more likely to adhere to an UNSUP program compared to a SUP in the long term because UNSUP programs are easier to integrate into their lives [57]. Nevertheless, other factors associated with SUP might also be of relevance, such as obtaining direct feedback from a professional, the social component of being with other participants, or having greater material resources. Further research is therefore needed to confirm the role of attendance of SUP versus UNSUP in the long term in addition to studies that analyze adherence to training rather than only attendance rates.

### Effectiveness of Supervised Exercise Intervention (SUP) Versus Unsupervised Exercise Intervention (UNSUP)

Our findings based on preliminary meta-analytical evidence suggest that SUP could provide greater benefits compared to UNSUP in different physical functions (i.e., knee extension strength, STS, TUG, usual gait speed, and lean mass) and well-being (i.e., HRQoL) measures. In line with our results, the meta-analysis of Lacroix et al. [13] found that SUP could provide additional benefits on some strength/power and balance measures. However, most of our findings became non-significant after sensitivity analyses, with the exception of knee extension strength. The observed improvement in knee extension strength is potentially relevant, as this outcome has proven to be critical for preventing osteopenia or osteoporosis [58]. Knee extension strength is also an important predictor of functional performance in older adults, as it is essential for activities of daily living and general well-being [59]. Remarkably, a systematic review and meta-analysis including data from two million adults concluded that higher levels of knee extension strength were associated with a lower risk of mortality, regardless of age and follow-up period [60].

On the other hand, no significant benefits were found for the remaining physical function outcomes (i.e.*,* handgrip, FRT, one leg stance, balance scales, tandem stance, maximum gait speed, 6-min walk test, maximal oxygen uptake, body mass index, body mass, and body fat). There are different hypotheses that may partially explain the lack of benefits obtained. Firstly, participants will improve to a greater extent what they specifically train in their workouts (e.g., if most training sessions include lower-body exercises such as the Otago Exercise Program [37], participants will improve more in outcomes such as knee extension or STS since exercises with similar movement patterns are included). Secondly, it is possible that significant improvements will only be observed in those outcomes in which participants show more possibility of improvement because they have a lower starting level. This is consistent with the results obtained since, as we age, we tend to lose power, strength, and muscle mass due to the natural phenomenon of sarcopenia [61], so participants may be more likely to improve outcomes such as knee extension strength or STS if their baseline level is low. Lastly, there was large heterogeneity in the characteristics of the included studies and the applied interventions, as well as some potentially confounding factors that may influence the lack of additional benefits observed.

### Confounding Factors in SUP and UNSUP Exercise Interventions

Of note, in many studies the exercise intervention applied in SUP and UNSUP differed substantially. We observed that training variables (i.e., volume, frequency, intensity, and type of exercise) were overall better reported in the SUP group than in the UNSUP group, which hinders drawing strong conclusions on the influence of these factors. In line with previous systematic reviews and meta-analyses [12, 62], a higher exercise intensity was usually applied in SUP than in UNSUP. For example, Iliffe et al. [37] reported that the SUP group trained at a higher intensity than the UNSUP group, and in the study by Watson et al. [14], the SUP group trained using weights equivalent to > 80–85% repetition maximum (RM) while the UNSUP group trained using a lower intensity (< 60% RM). This may be due to the fact that the target population are older people, which might lead professionals to be more conservative when prescribing intensities for the UNSUP group to avoid the potential risk of adverse events (e.g.*,* injuries, falls) or to the participants themselves self-selecting a lower intensity during UNSUP. In a few studies the exercise selection for the SUP group was similar to the UNSUP group, taking into account the limitations and advantages in terms of facilities and equipment involved when training at a center versus training at home [40, 43]. Additionally, in most studies the type of exercise intervention was not comparable between groups. For example, Cecchi et al. [32] compared a SUP multicomponent physical exercise program (i.e., strength, balance, aerobic, and stretching) versus an UNSUP program consisting solely of regular walking (only aerobic). To account for this issue, a sub-analysis was performed comparing those studies that equated the exercise intervention as closely as possible (i.e., similar volume, frequency, intensity, and type of exercise) between the SUP and UNSUP groups. Significant differences were found for knee extension strength in those studies that performed a similar exercise program in both groups. However, the number of studies included in each of the 12 analyzed outcomes ranged from three to nine, and the remaining six outcomes could not be analyzed due to the low number of studies available.

Moreover, in some cases participants in the SUP or the UNSUP groups performed only part of the exercise sessions with or without supervision, respectively (only 13/34 studies did not include any supervised sessions during the intervention). Rarely, the two exercise groups only differed in the amount of guidance they received [30, 49]. Therefore, we conducted a second sub-analysis comparing those studies in which participants performed more than two-thirds of the sessions in the assigned condition (i.e., the UNSUP group performed at least 66% of the sessions without supervision) and significant differences in knee extension strength, STS, FRT, TUG, usual gait speed, lean mass, and HRQoL were observed. Eighteen outcomes could be analyzed in this sub-analysis, but the number of studies included in each outcome was reduced (from three to 13 studies). These limitations derived from the reduced number of studies included in both sub-analyses make it difficult to reach definitive conclusions.

Future research should examine the safety, attendance/adherence rates, and effectiveness of SUP versus UNSUP focusing on comparable training parameters, including volume, frequency, intensity, and exercise modality. These studies should specifically compare programs that differ solely in the presence or absence of supervision (i.e., fully supervised vs. fully unsupervised). This approach will provide valuable insights into the potential benefits and limitations associated with supervision, shedding light on the optimal design of exercise interventions for various populations.

### Practical Implications

Our results have shown that SUP could provide additional benefits to UNSUP on some specific outcomes. The reason why UNSUP may not be as effective as SUP for improving some outcomes might be partly due to the fact that workouts conducted under the supervision of a professional may be performed with a higher quality. For example, SUP usually trains with better technical execution of the exercises, higher intensity and rating of perceived effort, better implementation of individualization and progression principles, and higher motivation due to direct feedback resulting in greater improvements [63, 64]. Therefore, given its potential superiority, SUP might be recommended over UNSUP when possible. However, there are some barriers usually associated with SUP in this population. For example, a systematic review conducted in the oldest old (i.e., people aged 80 years and over) showed that some of the main limitations to exercise identified were costs, transport, lack of access to exercise facilities, no exercise companion or being alone, care of siblings or others, fatigue, and embarrassment [65]. In addition, Costello et al. [66] reported lack of time and discipline, potential for injury, inadequate motivation, boredom and intimidation as main barriers to regular physical activity.

Previous meta-analyses have shown that UNSUP is effective for improving health-related outcomes in older adults [7, 10, 67]. Our research group showed that, compared with no exercise, UNSUP could be safe and effective for improving measures of muscle strength/power and balance in community-dwelling older adults, although the adherence to these programs was low [9]. Similarly, a meta-analysis including 17 studies also reported that UNSUP was effective for enhancing physical fitness in healthy older adults [10]. More recently, a meta-analysis including 12 studies (performed in both adolescents and adults) concluded that supervised resistance training could provide small additional benefits over unsupervised training on muscle strength, but no consistent differences were found for body composition [12]. Thus, when SUP is not feasible, UNSUP could be a safe and cost-effective alternative for improving the fitness and health of older adults.

### Strengths and Limitations

One of the main strengths of the present study is that it provides novel information, as we focused solely on older adults, including a large number of studies (34 RCTs and 2830 participants), and analyzed both physical function and well-being outcomes. Another major strength is that previous systematic reviews and meta-analyses have often focused on a single specific type of exercise (e.g., strength or balance alone) whereas the present review included studies that examined all exercise types (i.e., strength, balance, flexibility, aerobic, or a combination thereof). Conversely, some limitations of the present study should be acknowledged. Notably, the low number of available studies for some of the meta-analyzed outcomes made the conclusions preliminary. One potential confounding factor is that most studies did not equate all training variables (i.e., volume, frequency, intensity, and type of exercise) for the SUP and UNSUP groups. To account for this issue, we performed sub-analyses comparing those studies that performed a similar exercise intervention in both groups. The lack of a consistent terminology regarding the degree of supervision in exercise interventions can be considered another confounding factor, since most of the UNSUP programs included some supervised sessions. Therefore, we performed additional sub-analyses defining an objective concept of training supervision (≥ 66% supervised sessions in the SUP group and ≥ 66% unsupervised sessions in the UNSUP group). There was also a lack of homogeneity in the tests used for assessment, making it difficult to reach definitive conclusions due to the small number of studies included in each meta-analyzed outcome. Future studies should take all these limitations into consideration. Finally, it is worth noting that the included studies analyzed both healthy and diseased populations, but given the heterogeneity of the populations assessed within- and between-studies, and the low number of studies included, we were unable to perform sub-analyses to determine how participants’ characteristics (i.e., healthy vs. clinical populations) moderate exercise benefits. Indeed, given the advanced age of the participants included, setting an objective definition of “healthy” is highly complex, since most participants presented some comorbidities (e.g., diabetes, hypertension, obesity, osteoarthritis, sarcopenia, frailty).

## Conclusion

**Fig. 2:**
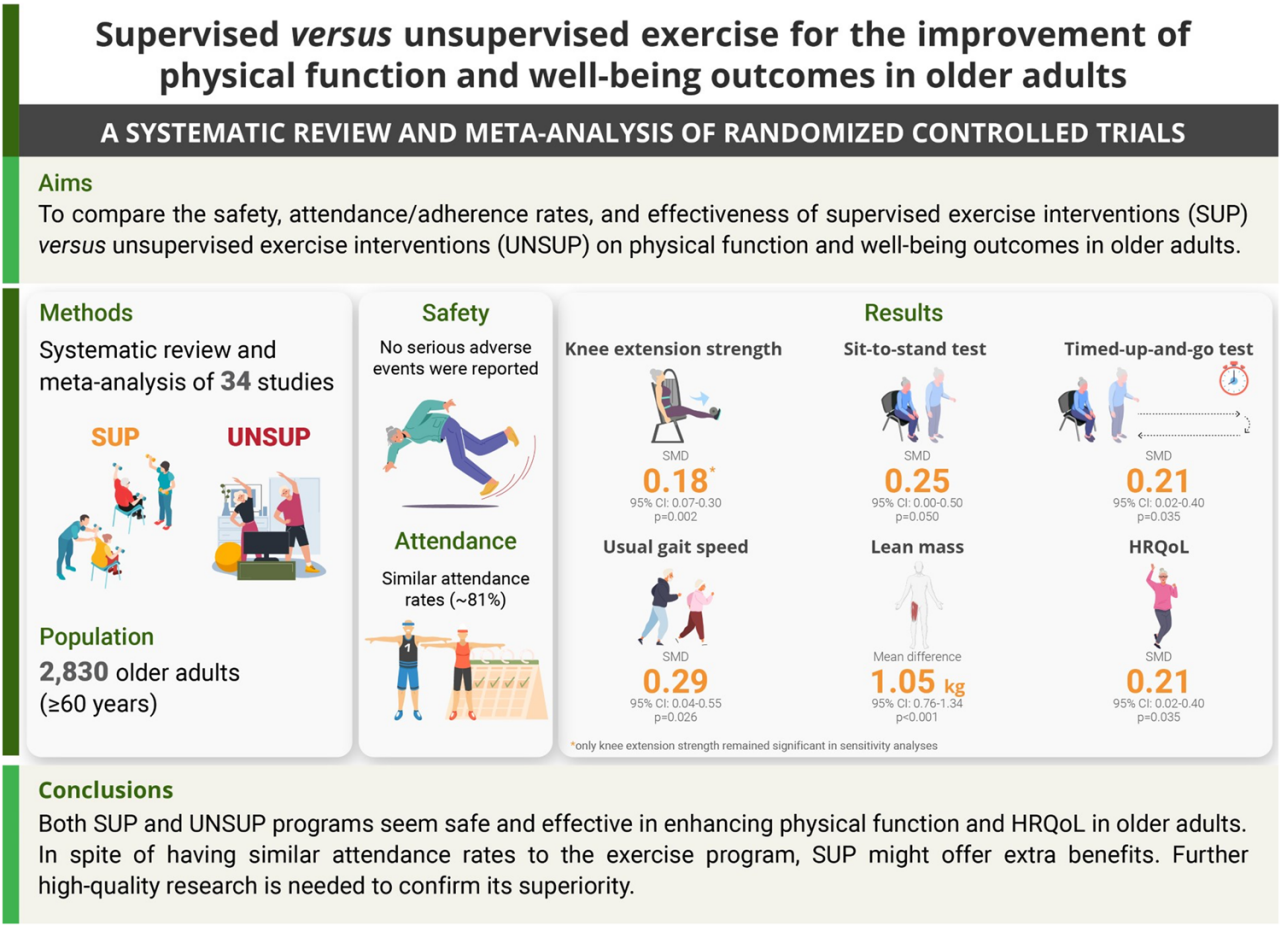
Graphical summary of the study findings. *CI* confidence interval, *HRQoL* health-related quality of life, *SMD* standardized mean difference, *SUP* supervised exercise interventions, *UNSUP* unsupervised exercise interventions

The present study suggests that SUP may offer certain advantages over UNSUP in enhancing physical function and well-being outcomes among older adults. Nevertheless, given that both interventions show high attendance rates and similar levels of safety, UNSUP appears to be an accessible approach for older adults, which might overcome some of the limitations associated with SUP. Future research should aim to examine the safety, attendance/adherence rates, and effectiveness of SUP versus UNSUP, focusing on equating training parameters between the two groups and differing only in the presence or absence of supervision (i.e., the UNSUP group cannot include supervised sessions or vice versa). These studies will provide valuable information about the benefits and limitations of supervision, informing the optimal design of exercise interventions. Figure 2 summarizes the findings obtained in this research.

### Supplementary Information

Below is the link to the electronic supplementary material.

Supplementary file1 (PDF 2800 KB)

## Declarations

### Availability of Data and Materials

All data generated or analyzed during this study are included in this published article and its Supplementary files.
