# Supplementary material for: Supervised Versus Unsupervised Exercise for the Improvement of Physical Function and Well-Being Outcomes in Older Adults: A Systematic Review and Meta-analysis of Randomized Controlled Trials
Source: Sports Med. 2024 Apr 22;54(7):1877–906. doi: 10.1007/s40279-024-02024-1 (PMC11258164; doi:10.1007/s40279-024-02024-1)
Supplement: Supplementary file 1 — Supplementary file1 (PDF 2800 KB) [file 40279_2024_2024_MOESM1_ESM.pdf]

Electronic Supplementary Material

Article title: Supervised *versus* unsupervised exercise for the improvement of physical function and well-being outcomes in older adults: A systematic review and meta-analysis of randomized controlled trials

Journal name: Sports Medicine

Authors names:

Paola Gómez-Redondo[a,b,c] MSc, Pedro L. Valenzuela[d,e] PhD, Javier S. Morales[f,g] PhD, Ignacio Ara[a,b,c] PhD, Asier Mañas[a,b,c,h,i*] PhD

Affiliations:

[a] GENUD Toledo Research Group, Faculty of Sports Sciences, Universidad de Castilla-La Mancha, Toledo, Spain.

[b] CIBER on Frailty and Healthy Aging, Instituto de Salud Carlos III, Madrid, Spain.

[c] Instituto de Investigación Sanitaria de Castilla-La Mancha (IDISCAM), Junta de Comunidades de Castilla-La Mancha (JCCM), Toledo, Spain.

[d] Physical Activity and Health Research Group (PaHerg), Research Institute of Hospital 12 de Octubre (imas12), Madrid, Spain.

[e] Biology Systems Department, University of Alcalá, Madrid.

[f] MOVE-IT Research Group, Department of Physical Education, Faculty of Education Sciences, University of Cadiz, Cadiz, Spain.

[g] Biomedical Research and Innovation Institute of Cádiz (INiBICA) Research Unit, Puerto Real University Hospital, University of Cadiz, Cadiz, Spain.

[h] Center UCM-ISCIII for Human Evolution and Behavior, 28029 Madrid, Spain.

[I] Faculty of Education, Complutense University of Madrid, 28040 Madrid, Spain.

[*]Address correspondence to Asier Mañas, GENUD Toledo Research Group, Universidad de Castilla-La Mancha, Avda. Carlos III s/n, 45071, Toledo, Spain.

E-mail address: asier.manas@uclm.es (Asier Mañas)

Study name	Std diff in means	Lower limit	Upper limit	p-Value	Relative weight
Bieler et al. 2018	0.18	-0.21	0.57	0.373	9.17
Boshuizen et al. 2005	0.32	-0.38	1.01	0.376	2.85
Brown et al. 2000	0.23	-0.20	0.67	0.289	7.38
Costa et al. 2020	0.63	-0.17	1.44	0.124	2.12
Donat et al. 2007	0.21	-0.48	0.91	0.552	2.86
Morrison et al. 2018	0.70	0.06	1.34	0.033	3.34
Nai-Hsin et al. 2020	-0.11	-0.43	0.22	0.524	13.16
Tsekoura et al. 2018	0.42	-0.24	1.08	0.210	3.18
Opdenacker et al. 2011	0.28	-0.08	0.64	0.132	10.73
Watanabe et al. 2020	0.15	-0.02	0.33	0.087	45.18
	0.18	0.07	0.30	0.002	

Heterogeneity: $Tau^2 = 0.00$; $Q=7.786$ (df=9), $p=0.556$; $I^2 = 0\%$

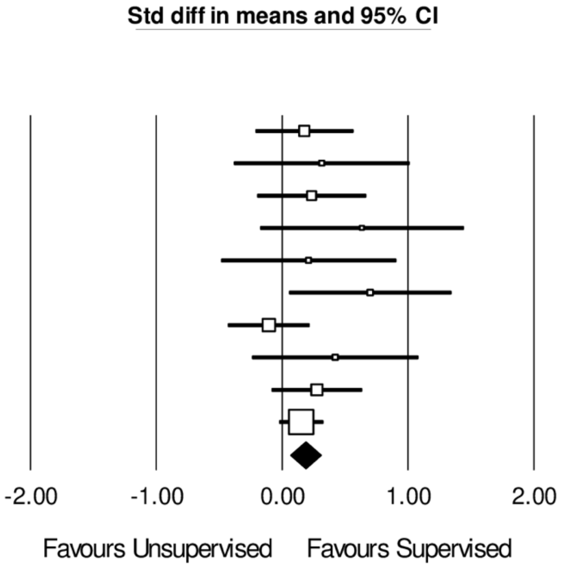

Supplementary Figure S1. Effects of supervised exercise interventions *versus* unsupervised exercise interventions on knee extension strength in older adults.

Study name	Statistics for each study				
	Std diff in means	Lower limit	Upper limit	p-Value	Relative weight
Bieler et al. 2018	0.15	-0.24	0.54	0.449	22.1
Brown et al. 2000	0.51	0.07	0.95	0.022	19.6
Costa et al. 2020	0.11	-0.68	0.90	0.780	8.9
Morrison et al. 2018	-0.21	-0.84	0.42	0.519	12.5
Nai-Hsin et al. 2020	0.05	-0.28	0.37	0.768	25.8
Tsekoura et al. 2018	0.91	0.22	1.59	0.010	11.1
	0.23	-0.04	0.50	0.089	

Heterogeneity: $Tau^2 = 0.044$; Q=8.580 (df=5), *p*=0.127; $I^2 = 41.7\%$

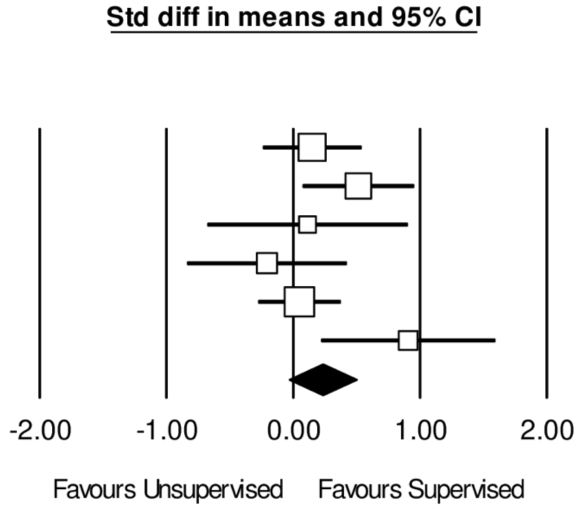

Supplementary Figure S2. Effects of supervised exercise interventions *versus* unsupervised exercise interventions on knee flexion strength in older adults.

Study name	Std diff in means	Lower limit	Upper limit	p-Value	Relative weight
Bieler et al. 2017	-0.07	-0.48	0.34	0.736	9.2
Costa et al. 2020	0.27	-0.52	1.06	0.502	5.5
Cyarto et al. 2008 B	0.07	-0.31	0.46	0.708	9.5
Iliffe et al. 2014	0.00	-0.18	0.18	1.000	11.5
Lacroix et al. 2015	1.03	0.36	1.70	0.003	6.5
Nai-Hsin et al. 2020	0.20	-0.13	0.52	0.238	10.1
Perez-Dominguez et al. 2021	0.21	-0.31	0.74	0.422	8.0
Sandberg et al. 2022	0.12	-0.30	0.54	0.573	9.1
Tsekoura et al. 2018	0.56	-0.11	1.23	0.099	6.6
Opdenacker et al. 2011	-0.46	-0.82	-0.10	0.013	9.7
Watanabe et al. 2020	0.11	-0.07	0.28	0.230	11.5
Watson et al. 2015	4.23	2.89	5.57	0.000	2.8
	0.25	-0.00	0.50	0.050	

Heterogeneity: $Tau^2 = 0.136$; $Q=57.592$ ($df=11$), $p<0.001$; $I^2 = 80.9\%$

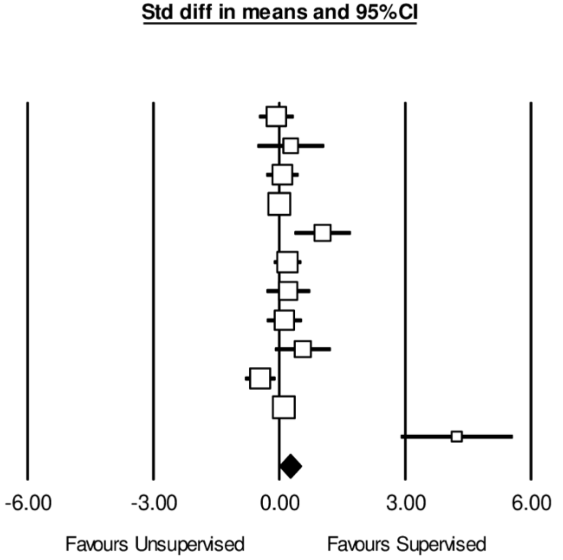

Supplementary Figure S3. Effects of supervised exercise interventions *versus* unsupervised exercise interventions on sit-to-stand test in older adults.

Study name	Statistics for each study				
	Std diff in means	Lower limit	Upper limit	p-Value	Relative weight
Nai-Hsin et al. 2020	-0.15	-0.47	0.18	0.372	29.1
Perez-Dominguez et al. 2021	0.23	-0.29	0.75	0.393	19.6
Tsekoura et al. 2018	0.95	0.26	1.64	0.007	14.1
Watanabe et al. 2020	0.00	-0.17	0.17	1.000	37.2
	0.13	-0.18	0.45	0.404	

Heterogeneity: $Tau^2 = 0.062$; $Q=8.645$ $(df=3)$, $p=0.034$; $I^2 = 65.3\%$

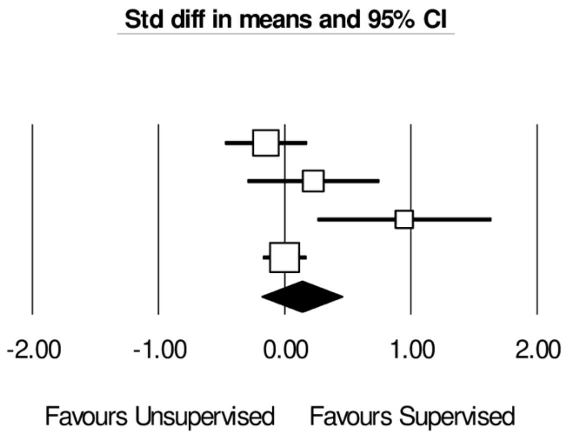

Supplementary Figure S4. Effects of supervised exercise interventions *versus* unsupervised exercise interventions on handgrip strength in older adults.

Study name	Std diff in means	Lower limit	Upper limit	p-Value	Relative weight
	**Statistics for each study**				
Brown et al. 2000	0.00	-0.43	0.43	1.000	19.4
Iliffe et. 2014	0.02	-0.16	0.20	0.817	22.3
Lacroix et al. 2015	0.87	0.22	1.52	0.009	16.2
Lidenmann et al. 2004	-0.35	-1.21	0.52	0.431	13.2
Watanabe et al. 2020	0.02	-0.16	0.19	0.848	22.4
Watson et al. 2015	5.31	3.73	6.90	0.000	6.5
	0.45	-0.03	0.93	0.064	

Heterogeneity: $Tau^2 = 0.257$; $Q=49.727$ (df=5), $p<0.001$; $I^2 = 89.9\%$

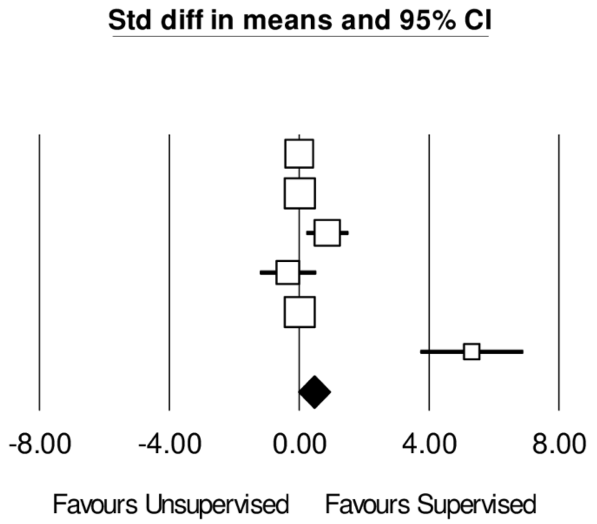

Supplementary Figure S5. Effects of supervised exercise interventions *versus* unsupervised exercise interventions on functional reach test in older adults.

Study name	Std diff in means	Lower limit	Upper limit	p-Value	Relative weight
Brown et al. 2000	0.66	0.21	1.10	0.004	20.7
Cyarto et al. 2008 A	0.60	0.21	0.99	0.003	21.9
Donat et al. 2007	0.10	-0.60	0.79	0.787	15.2
Nai-Hsin et al. 2020	-0.12	-0.44	0.21	0.475	23.4
Perez-Dominguez et al. 2021	-0.33	-0.86	0.19	0.212	18.8
	0.19	-0.21	0.59	0.351	

Heterogeneity: $Tau^2 = 0.152$; Q=15.83 (df=4), p=0.003; $I^2 = 74.7\%$

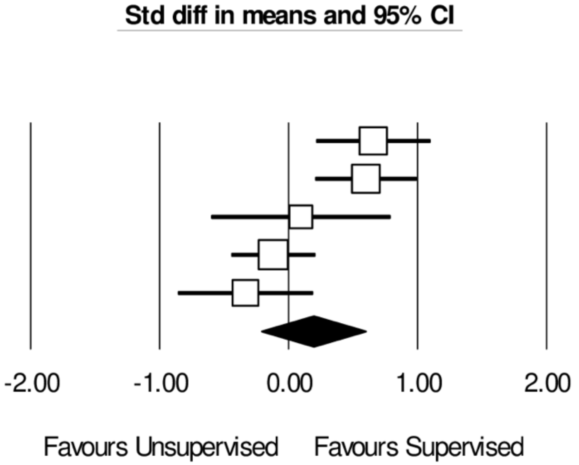

Supplementary Figure S6. Effects of supervised exercise interventions *versus* unsupervised exercise interventions on one leg stance in older adults.

Study name	Std diff in means	Lower limit	Upper limit	p-Value	Relative weight
Almeida et al. 2013	0.05	-0.51	0.61	0.853	19.4
Brown et al. 2000	0.49	0.05	0.92	0.030	20.8
Cyarto et al. 2008 A	-0.47	-0.86	-0.08	0.017	21.3
Donat et al. 2007	0.20	-0.50	0.90	0.574	17.8
Karahan et al. 2015	1.14	0.69	1.58	0.000	20.7
	0.28	-0.32	0.88	0.356	

(Statistics for each study)

Heterogeneity: $Tau^2 = 0.336$; $Q=29.974$ (df=4), $p<0.001$; $I^2 = 86.7\%$

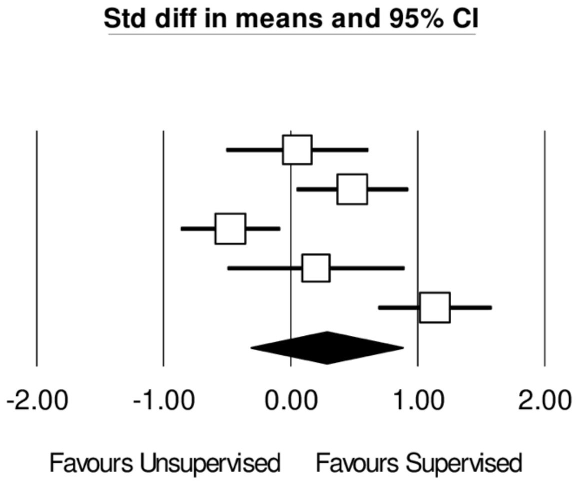

Supplementary Figure S7. Effects of supervised exercise interventions *versus* unsupervised exercise interventions on balance scales in older adults.

Study name	Statistics for each study				
	Std diff in means	Lower limit	Upper limit	p-Value	Relative weight
Brown et al. 2000	-0.52	-1.10	0.05	0.073	26.8
Donat et al. 2007	-0.05	-0.74	0.64	0.888	23.1
Lacroix et al. 2015	0.60	-0.02	1.23	0.059	25.2
Morrison et al. 2018	0.38	-0.25	1.02	0.236	24.9
	0.10	-0.42	0.61	0.715	

Heterogeneity: $Tau^2 = 0.170$; $Q=7.959$ (df=3), $p=0.047$; $I^2 = 62.3\%$

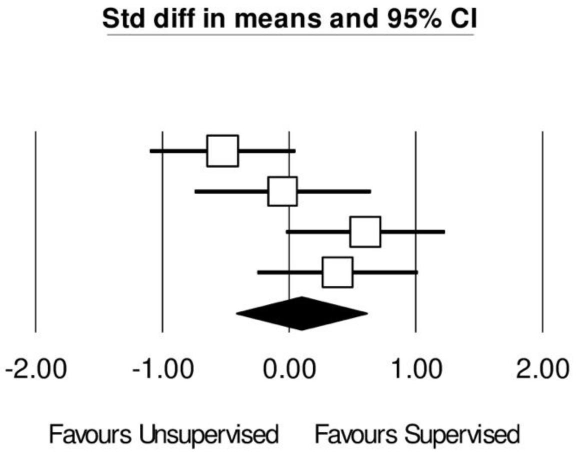

Supplementary Figure S8. Effects of supervised exercise interventions *versus* unsupervised exercise interventions on tandem stance with eyes closed in older adults.

**Study name**	**Statistics for each study**				
	Std diff in means	Lower limit	Upper limit	p-Value	Relative weight
Boshuizen et al. 2005	-0.67	-1.38	0.04	0.064	23.3
Cyarto et al. 2008 A	0.50	0.11	0.89	0.013	28.9
Donat et al. 2007	-0.60	-1.31	0.11	0.095	23.3
Morrison et al. 2018	0.60	-0.04	1.24	0.068	24.6
	-0.01	-0.66	0.65	0.983	

Heterogeneity: $Tau^2 = 0.345$; Q=13.348 (df=3), p=0.002; $I^2 = 79.1\%$

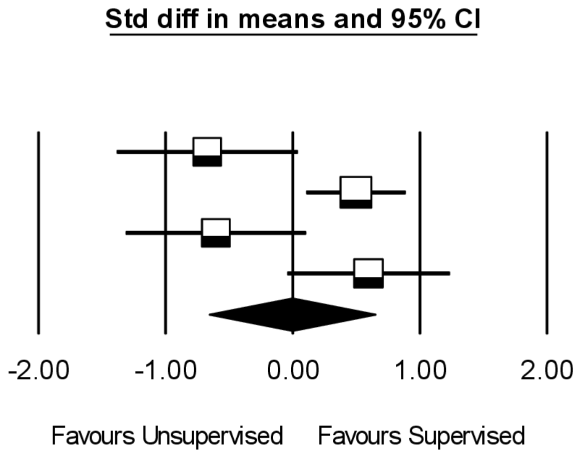

Supplementary Figure S9. Effects of supervised exercise interventions *versus* unsupervised exercise interventions on tandem stance with eyes open in older adults.

Study name	Std diff in means	Lower limit	Upper limit	p-Value	Relative weight
Almeida et al. 2013	0.03	-0.53	0.58	0.929	6.0
Bieler et al. 2017	-0.08	-0.49	0.33	0.693	7.8
Boshuizen et al. 2005	0.20	-0.50	0.89	0.574	4.7
Cyarto et al. 2008	0.00	-0.39	0.39	1.000	8.2
Donat et al. 2007	0.18	-0.52	0.88	0.613	4.7
Ebersbach et al. 2010	-0.73	-1.38	-0.08	0.027	5.1
Iliffe et al. 2014	0.02	-0.18	0.21	0.867	10.7
Karahan et al. 2015	0.05	-0.36	0.46	0.810	7.8
Lacroix et al. 2015	0.88	0.23	1.53	0.008	5.1
Nai-Hsin et al. 2020	0.27	-0.06	0.59	0.106	9.0
Perez-Dominguez et al. 2021	0.48	-0.05	1.00	0.076	6.4
Tsekoura et al. 2018	0.37	-0.29	1.03	0.266	5.0
Watanabe et al. 2020	0.22	0.05	0.40	0.012	11.0
Watson et al. 2015	2.65	1.63	3.67	0.000	2.8
Wu et al. 2010	0.20	-0.41	0.81	0.523	5.5
	0.21	0.01	0.40	0.035	

Heterogeneity: Tau² = 0.082; Q=42.192 (df=14), *p*<0.001; I² = 66.8%

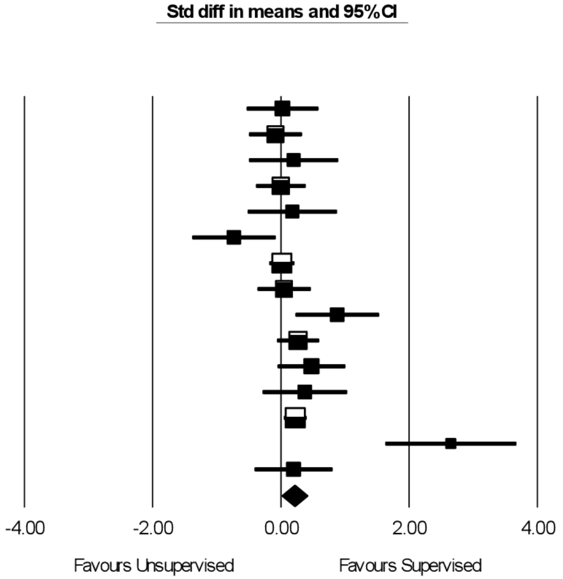

Supplementary Figure S10. Effects of supervised exercise interventions *versus* unsupervised exercise interventions on timed-up-and-go test in older adults.

Study name	Statistics for each study				
	Std diff in means	Lower limit	Upper limit	p-Value	Relative weight
Almeida et al. 2013	0.55	-0.01	1.12	0.056	11.7
Boshuizen et al. 2005	0.36	-0.34	1.06	0.312	9.1
Brown et al. 2000	0.06	-0.37	0.50	0.775	15.4
Costa et al. 2020	0.00	-0.79	0.79	1.000	7.6
Ebersbach et al. 2010	0.69	0.05	1.34	0.035	10.0
Perez-Dominguez et al. 2021	0.00	-0.52	0.52	1.000	12.9
Tsekoura et al. 2018	1.15	0.45	1.86	0.001	8.9
Watanabe et al. 2020	0.05	-0.12	0.22	0.568	24.3
	0.29	0.03	0.55	0.026	

Heterogeneity: Tau² = 0.063; Q=14.643 (df=7), p=0.041; I² = 52.2%

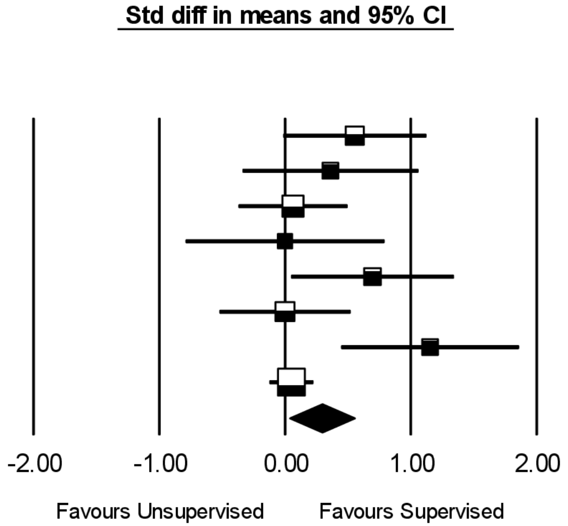

Supplementary Figure S11. Effects of supervised exercise interventions *versus* unsupervised exercise interventions on usual gait speed in older adults.

Study name	Std diff in means	Lower limit	Upper limit	p-Value	Relative weight
Brown et al. 2000	-0.01	-0.44	0.42	0.957	15.9
Costa et al. 2020	1.00	0.16	1.84	0.019	5.4
Hinman et al. 2002	-0.09	-0.61	0.42	0.728	12.3
Lidenmann et al. 2004	0.64	-0.24	1.52	0.154	5.0
Nai-Hsin et al. 2020	0.05	-0.28	0.37	0.772	22.9
Watanabe et al. 2020	0.23	0.06	0.41	0.010	38.4
	0.17	-0.03	0.38	0.100	

(Statistics for each study)

Heterogeneity: $Tau^2 = 0.021$; Q=7.529 (df=5), *p*=0.184; $I^2 = 33.6\%$

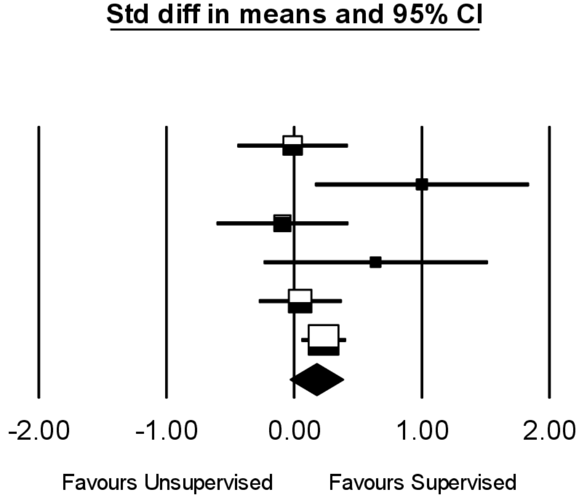

Supplementary Figure S12. Effects of supervised exercise interventions *versus* unsupervised exercise interventions on maximum gait speed in older adults.

Study name	Statistics for each study				
	Difference in means	Lower limit	Upper limit	p-Value	Relative weight
Bieler et al. 2017	-7.00	-34.17	20.17	0.614	24.0
Gardner et al. 2014	29.00	0.39	57.61	0.047	22.6
Nai-Hsin et al. 2020	-10.57	-40.79	19.65	0.493	21.1
Perez-Dominguez et al. 2021	20.10	-28.78	68.98	0.420	10.3
Sandberg et al. 2022	-13.20	-42.39	15.99	0.375	22.0
	1.80	-15.47	19.08	0.838	

Heterogeneity: Tau² = 130.992; Q=6.067 (df=4), *p*=0.194; I² = 34.1%

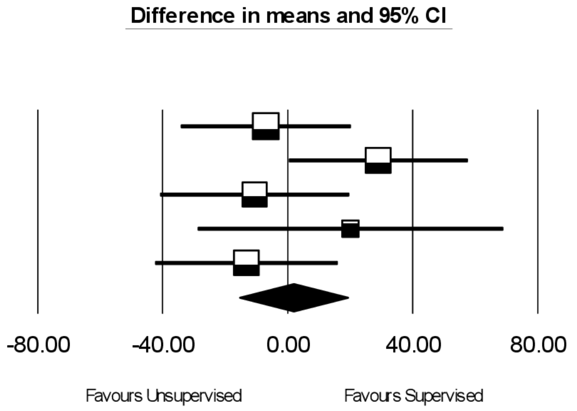

Supplementary Figure S13. Effects of supervised exercise interventions *versus* unsupervised exercise interventions on six-minute walk test in older adults.

Study name	Statistics for each study				
	Difference in means	Lower limit	Upper limit	p-Value	Relative weight
Gardner et al. 2011	-0.30	-1.57	0.97	0.643	56.9
Opdenacker et al. 2011	1.30	-0.33	2.93	0.118	39.1
Sian et al. 2022	-0.42	-6.14	5.30	0.886	3.9
	0.32	-0.83	1.47	0.583	

Heterogeneity: Tau² = 0.185; Q=2.366 (df=2), *p*=0.306; I² = 15.5%

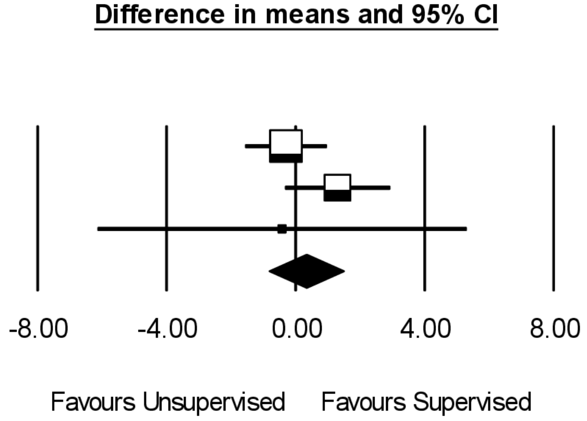

Supplementary Figure S14. Effects of supervised exercise interventions *versus* unsupervised exercise interventions on maximal oxygen uptake in older adults.

Study name	Statistics for each study				
	Difference in means	Lower limit	Upper limit	p-Value	Relative weight
Nai-Hsin et al. 2020	0.10	-1.29	1.49	0.888	25.2
Tsekoura et al. 2018	-0.04	-1.26	1.18	0.949	32.7
Opdenacker et al. 2011	-0.10	-1.17	0.97	0.855	42.2
	-0.03	-0.73	0.67	0.933	

Heterogeneity: Tau² = 0.00; Q=0.050 (df=2), *p*=0.975; I² = 0%

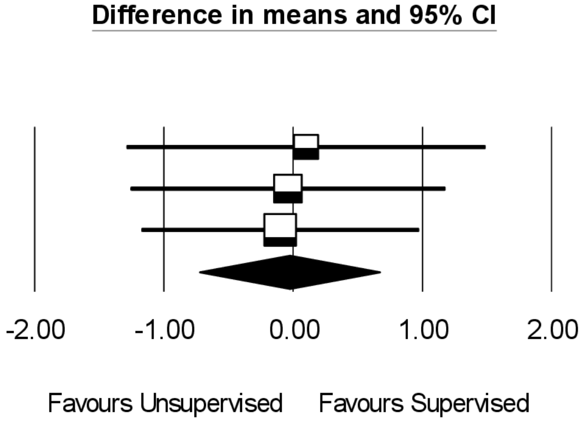

Supplementary Figure S15. Effects of supervised exercise interventions *versus* unsupervised exercise interventions on body mass index in older adults.

Study name	Difference in means	Lower limit	Upper limit	p-Value	Relative weight
Bittar et al. 2006	-1.20	-7.62	5.22	0.714	0.4
Nai-Hsin et al. 2020	0.27	-2.48	3.02	0.847	2.3
Watson et al. 2015	-0.37	-0.79	0.05	0.087	97.3
	-0.36	-0.78	0.06	0.092	

Heterogeneity: Tau² = 0.00; Q=0.270 (df=2), *p*=0.874; I² = 0%

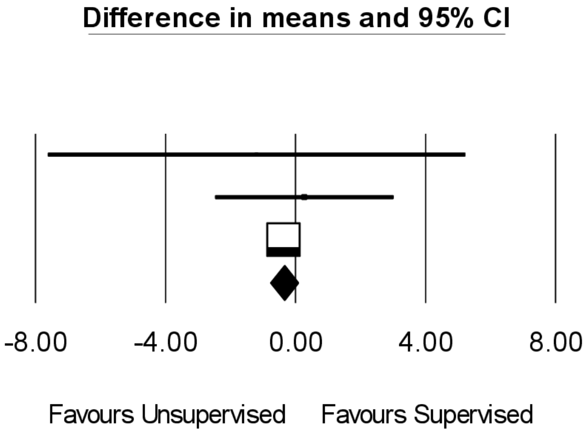

Supplementary Figure S16. Effects of supervised exercise interventions *versus* unsupervised exercise interventions on body mass in older adults.

Study name	Statistics for each study				
	Std diff in means	Lower limit	Upper limit	p-Value	Relative weight
Bittar et al. 2015	0.00	-0.67	0.67	1.000	12.6
Nai-Hsin et al. 2020	0.08	-0.24	0.41	0.620	37.2
Opdenacker et al. 2011	0.02	-0.34	0.38	0.916	33.0
Watson et al. 2015	0.99	0.19	1.78	0.015	9.5
Sian et al. 2022	0.03	-0.85	0.90	0.955	7.9
	0.13	-0.13	0.39	0.314	

Heterogeneity: Tau² = 0.019; Q=5.110 (df=4), *p*=0.276; I² = 21.7%

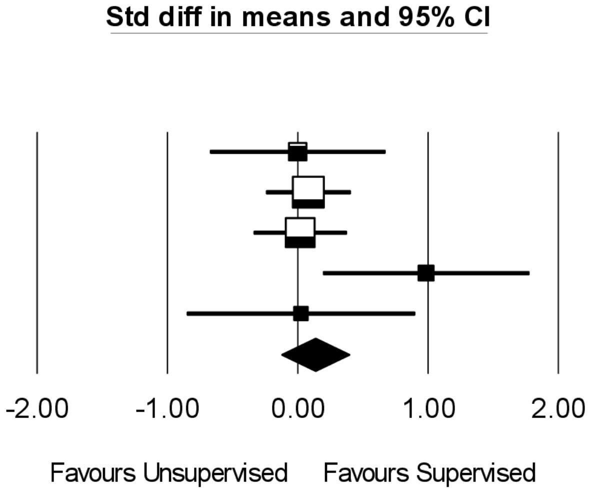

Supplementary Figure S17. Effects of supervised exercise interventions *versus* unsupervised exercise interventions on body fat in older adults.

Study name	Difference in means	Lower limit	Upper limit	p-Value	Relative weight
Bittar et al. 2015	0.20	-2.35	2.75	0.878	1.3
Tsekoura et al. 2018	0.59	-1.78	2.96	0.625	1.5
Watson et al. 2015	1.07	0.77	1.37	0.000	97.0
Sian et al. 2022	0.29	-6.90	7.48	0.937	0.2
	1.05	0.76	1.34	0.000	

Heterogeneity: Tau² = 0.00; Q=0.631 (df=3), p=0.889; I² = 0%

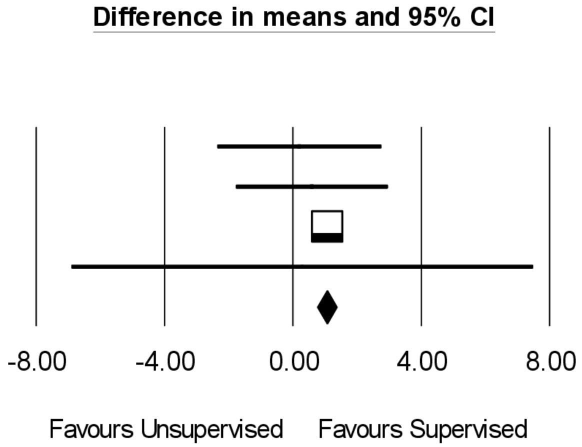

Supplementary Figure S18. Effects of supervised exercise interventions *versus* unsupervised exercise interventions on lean mass in older adults.

Study name	Std diff in means	Lower limit	Upper limit	p-Value	Relative weight
Ebersbach et al. 2010	0.30	-0.33	0.93	0.356	6.6
Kakkos et al. 2005	-1.10	-2.00	-0.20	0.017	3.8
Iliffe et al. 2014	0.00	-0.17	0.17	1.000	19.6
Mickle et al. 2016	0.28	-0.20	0.76	0.247	9.5
Perez-Dominguez et al. 2021	-0.08	-0.76	0.61	0.831	5.8
Tsekoura et al. 2018	0.42	-0.24	1.08	0.210	6.2
Bieler et al. 2017	0.40	0.18	0.62	0.000	18.0
Wu et al. 2010	0.36	-0.10	0.82	0.122	10.0
Karahan et al. 2015	0.35	0.21	0.50	0.000	20.6
	0.21	0.02	0.40	0.035	

Heterogeneity: Tau² = 0.041; Q=21.683 (df=8), *p*=0.006; I² = 63.1%

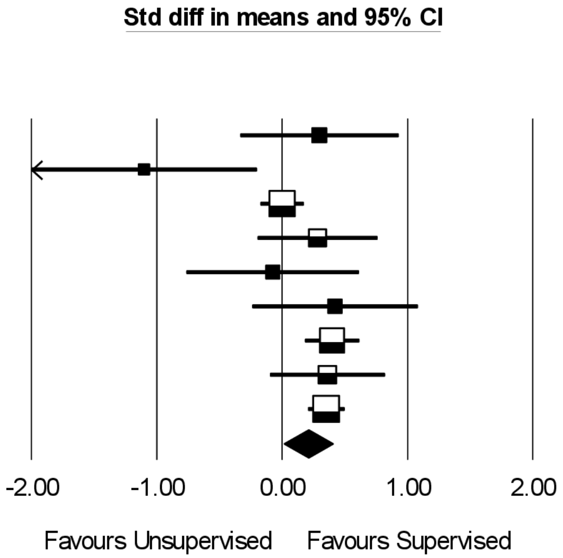

Supplementary Figure S19. Effects of supervised exercise interventions *versus* unsupervised exercise interventions on health-related quality of life in older adults.

Supplementary Table S1. Search strategy.

DATABASE	SEARCH STRATEGY	FILTERS
PubMed	(home-based[Title/Abstract] OR telehealth[Title/Abstract] OR unsupervised[Title/Abstract] OR nonsupervised[Title/Abstract]) AND (supervised[Title/Abstract] OR supervision[Title/Abstract] OR gym-based[Title/Abstract] OR center-based[Title/Abstract] OR centre-based[Title/Abstract] OR group-based [Title/Abstract]) AND (training[Title/Abstract] OR exercise[Title/Abstract] OR "physical activity"[Title/Abstract])	None
Web of Science	(home-based OR telehealth OR unsupervised OR nonsupervised) AND (supervised OR supervision OR gym-based OR center-based OR centre-based OR group-based) AND (training OR exercise OR "physical activity")	Type of document: “Articles”
CINAHL		Title (TI) or Abstract (AB)
SPORTDiscus		
APA PsycInfo		

Supplementary Table S2. Eligibility criteria based on the PICOS approach.

Category	Inclusion criteria	Exclusion criteria
**Population**	Conducted in older adults (age ≥60 years or, if not available, mean age ≥65 years).	Studies involving participants diagnosed with a recent injury or acute disease (e.g., stroke, fractures, surgery).
**Intervention**	Supervised (synchronous supervision) exercise intervention whether face-to-face or videocall format. When a different intervention is included in addition to exercise (nutritional, cognitive, psychological, etc.), it should be equal in both groups.	Rehabilitation interventions (e.g., after an acute event such as injuries or surgery).
**Comparator**	Unsupervised exercise intervention.	If the exercise intervention is supervised directly by a previously educated person (*e.g.*, a family member) or remotely (*e.g.*, by video call).
**Outcome**	Studies should assess at least one of the following health-related endpoints: muscle strength, balance, physical performance[a], body composition, or health-related quality of life. In addition, safety and attendance/adherence rates were registered as indicators of feasibility.	Absence of baseline and/or post-intervention data.
**Study design**	Parallel-group randomized controlled trials.	Other types of study design.

[a] Following the European consensus on definition and diagnosis of sarcopenia, muscle strength included measures such as handgrip strength, arm curl test, knee extension strength or sit-to-stand test (STS), whereas physical performance included measures of whole-body function related to locomotion, including tests such as gait speed, timed-up-and go test (TUG), 6-minute walk test (6MWT), or maximal exercise capacity [1].

1. Cruz-Jentoft, AJ, Bahat, G, Bauer, J, Boirie, Y, Bruyère, O, Cederholm, T, et al. Sarcopenia: revised European consensus on definition and diagnosis. *Age Ageing*. **2019**;*48(1)*:16-31.

Supplementary Table S3. Sub-analyses attending to those studies that applied a similar intervention in both groups and those studies in which participants performed ≥66% of the sessions in the assigned condition.

**Outcome**	Sub-analysis	Studies (participants)	SMD (95%CI)	*p*-value
**Muscle strength**				
Knee extension strength	Same	5 (n = 649)	0.21 (0.06, 0.37)	**0.008**
	Different	5 (n = 477)	0.15 (-0.03, 0.33)	0.109
	≥66%	9 (n = 623)	0.21 (0.05, 0.37)	**0.010**
	≤66%	1 (n = 503)	N/A	N/A
STS	Same	6 (n = 838)	0.35 (-0.10, 0.80)	0.129
	Different	6 (n = 907)	0.26 (-0.16, 0.68)	0.221
	≥66%	10 (n = 750)	0.37 (0.03, 0.72)	**0.033**
	≤66%	2 (n = 995)	0.05 (-0.56, 0.67)	0.486
Handgrip	Same	3 (n = 609)	0.30 (-0.19, 0.79)	0.227
	Different	1 (n = 146)	N/A	N/A
	≥66%	3 (n = 239)	0.28 (-0.31, 0.87)	0.351
	≤66%	1 (n = 516)	N/A	N/A
**Balance**				
Functional reach test	Same	3 (n = 572)	0.19 (-0.83, 1.21)	0.718
	Different	3 (n = 593)	1.14 (0.09, 2.20)	0.034
	≥66%	4 (n = 173)	0.82 (0.16, 1.49)	**0.015**
	≤66%	2 (n = 992)	N/A	N/A
One leg stance	Same	3 (n = 208)	0.14 (-0.49, 0.77)	0.663
	Different	2 (n = 230)	N/A	N/A
	≥66%	5 (n = 438)	0.19 (-0.21, 0.59)	0.351
	≤66%	0	N/A	N/A
Balance scales	Same	3 (n = 201)	-0.13 (-0.61, 0.36)	0.610
	Different	2 (n = 174)	N/A	N/A
	≥66%	5 (n = 375)	0.28 (-0.32, 0.88)	0.356
	≤66%	0	N/A	N/A
Tandem stance with eyes closed	Same	3 (n = 118)	N/A	N/A
	Different	1 (n = 84)	N/A	N/A

	≥66%	4 (n = 202)	0.30 (-1.86, 2.47)	0.784
	≤66%	0	N/A	N/A
Tandem stance with eyes open	Same	4 (n = 229)	-1.02 (-2.91, 0.88)	0.294
	Different	0	N/A	N/A
	≥66%	4 (n = 229)	-1.02 (-2.91, 0.88)	0.294
	≤66%	0	N/A	N/A
**Physical performance**				
TUG	Same	9 (n = 911)	0.27 (-0.01, 0.54)	0.055
	Different	6 (n = 800)	0.15 (-0.17, 0.46)	0.359
	≥66%	13 (n = 802)	0.24 (0.01, 0.48)	**0.044**
	≤66%	2 (n = 909)	N/A	N/A
Usual gait speed	Same	5 (n = 686)	0.34 (-0.01, 0.70)	0.060
	Different	3 (n = 148)	0.24 (-0.25, 0.74)	0.330
	≥66%	7 (n = 323)	0.37 (0.07, 0.68)	**0.017**
	≤66%	1 (n = 511)	N/A	N/A
Maximum gait speed	Same	3 (n = 586)	0.19 (-0.15, 0.54)	0.274
	Different	3 (n = 255)	0.17 (-0.19, 0.54)	0.358
	≥66%	5 (n = 334)	0.16 (-0.14, 0.47)	0.296
	≤66%	1 (n = 507)	N/A	N/A
6MWT	Same	2 (n = 147)	N/A	N/A
	Different	3 (n = 357)	0.05 (-0.25, 0.35)	0.752
	≥66%	5 (n = 504)	1.8 (-15.5, 19.1)	0.838
	≤66%	0	N/A	N/A
VO_{2peak}	Same	1 (n = 20)	N/A	N/A
	Different	2 (n = 182)	N/A	N/A
	≥66%	3 (n = 202)	0.32 (-0.83, 1.47)	0.583
	≤66%	0	N/A	N/A
**Body composition**				
BMI	Same	1 (n = 36)	N/A	N/A
	Different	2 (n = 266)	N/A	N/A
	≥66%	3 (n = 302)	-0.03 (-0.73, 0.67)	0.933
	≤66%	0	N/A	N/A

Body mass	Same	1 (n = 34)	N/A	N/A
	Different	2 (n = 174)	N/A	N/A
	≥66%	3 (n = 208)	-0.36 (-0.78, 0.06)	0.092
	≤66%	0	N/A	N/A
Body fat	Same	2 (n = 54)	N/A	N/A
	Different	3 (n = 294)	0.19 (-0.15, 0.53)	0.273
	≥66%	5 (n = 348)	0.13 (-0.13, 0.39)	0.314
	≤66%	0	N/A	N/A
Lean mass	Same	3 (n = 90)	0.09 (-0.32, 0.51)	0.660
	Different	1 (n = 28)	N/A	N/A
	≥66%	4 (n = 118)	1.05 (0.76, 1.34)	**<0.001**
	≤66%	0	N/A	N/A
**HRQoL**				
HRQoL	Same	4 (n = 152)	0.06 (-0.32, 0.44)	0.762
	Different	5 (n = 801)	0.26 (0.03, 0,49)	**0.029**
	≥66%	7 (n = 423)	0.35 (0.23, 0.47)	**<0.001**
	≤66%	2 (n = 530)	0.05 (-0.23, 0.14)	0.622

BMI: Body mass index; HRQoL: Health-related quality of life; STS: Sit-to-stand test; 6MWT: Six-minute walk test; TUG: Timed-up-and-go test; VO2peak: Maximal oxygen uptake. Results are shown as standardized mean difference (SMD) along with 95% confidence intervals (CI). Significant *p*-values are in **bold font**.

Supplementary Table S4. Sub-analysis of high-quality studies.

Outcome	Studies (participants)	Effect estimate (95%CI)	*p*-value	Quality (mean TESTEX score, range)
Muscle strength				
**Knee extension strength (SMD)**	8 (n = 996)	0.16 (0.04, 0.28)	**0.012**	10 (7-13)
**Knee flexion strength (SMD)**	4 (n = 309)	0.23 (-0.09, 0.54)	0.159	12 (11-13)
**STS (SMD)**	12 (n = 1,745)	0.25 (0.00, 0.50)	**0.050**	12 (7-14)
**Handgrip (SMD)**	4 (n = 755)	0.13 (-0.18, 0.45)	0.404	11 (7-13)
Balance				
**Functional reach test (SMD)**	5 (n = 1,081)	0.60 (0.03, 1.18)	**0.040**	11 (7-14)
**One leg stance (SMD)**	4 (n = 354)	0.07 (-0.36, 0.50)	0.747	12 (9-14)
**Balance scales (SMD)**	3 (n = 241)	0.29 (-0.78, 1.36)	0.600	10 (7-14)
**Tandem stance with eyes closed (SMD)**	2 (n = 72)	0.29 (-0.34, 0.93)	0.366	11 (9-12)
**Tandem stance with eyes open (SMD)**	3 (n = 183)	-0.22 (-1.07, 0.64)	0.618	10 (8-14)
Physical performance				
**TUG (SMD)**	14 (n = 1,661)	0.22 (0.02, 0.43)	**0.033**	11 (7-14)
**Usual gait speed (SMD)**	6 (n = 700)	0.32 (-0.02, 0.66)	0.068	10 (7-13)
**Maximum gait speed (SMD)**	5 (n = 757)	0.21 (-0.03, 0.46)	0.086	9 (7-13)
**6MWT (m)**	5 (n = 504)	1.8 (-15.5, 19.1)	0.838	12 (10-13)
**VO_2peak (ml/kg/min)**	3 (n = 202)	0.32 (-0.83, 1.47)	0.583	12 (10-14)
Body composition				
**BMI (kg/m^2)**	3 (n = 302)	-0.03 (-0.73, 0.67)	0.933	12 (10-13)
**Body mass (kg)**	3 (n = 208)	-0.36 (-0.78, 0.06)	0.092	12 (9-14)
**Body fat (SMD)**	5 (n = 348)	0.13 (-0.13, 0.39)	0.314	12 (9-14)
**Lean mass (kg)**	4 (n = 118)	1.05 (0.76, 1.34)	**<0.001**	12 (9-14)
HRQoL (SMD)	9 (n = 953)	0.21 (0.02, 0.40)	**0.035**	10 (7-13)

BMI: Body mass index; HRQoL: Health-related quality of life; STS: Sit-to-stand test; 6MWT: Six-minute walk test; TUG: Timed-up-and-go test; VO2peak: Maximal oxygen uptake. Results are shown as standardized mean difference (SMD) or absolute mean difference along with 95% confidence intervals (CI). A higher TESTEX score indicates better quality. Significant *p*-values are in **bold font.**
